# Associations of exercise snacks with sarcopenia and all-cause mortality: A national cohort study

**DOI:** 10.5114/biolsport.2026.163369

**Published:** 2026-07-27

**Authors:** Jiulong Song, Guoguo Zhao, Zihao Chen, Jian Fu

**Affiliations:** 1 College of Physical Education, Yangzhou University, Yangzhou, Jiangsu, 225127, China; 2 Changzhou Vocational Institute of Textile and Garment, Changzhou, Jiangsu, 213164, China

**Keywords:** Exercise snacks, Sarcopenia, Handgrip strength, All-cause mortality, NHANES

## Abstract

We aimed to examine the associations of exercise snacks (ES), defined as brief accumulated bouts of moderate-to-vigorous physical activity, with sarcopenia and all-cause mortality. We analyzed data from the 2011–2014 National Health and Nutrition Examination Survey (NHANES). Exercise snacks were quantified using two indices: MIMS-ES (average daily MIMS units accumulated during exercise snack bouts) and Time-ES (total daily minutes spent in exercise snack bouts). Survey-weighted regression models, restricted cubic spline (RCS) analyses, and survival analyses were used to examine the associations of exercise snacks with sarcopenia, handgrip strength, and all-cause mortality. Subgroup and sensitivity analyses were conducted to assess the robustness of the results. A total of 2,969 participants were included. In the fully adjusted models, each 1-unit increase in MIMS-ES and each additional minute of Time-ES were associated with 8% and 9% lower odds of sarcopenia, respectively. Higher levels of exercise snacks were also associated with greater handgrip strength. RCS analyses revealed an L-shaped association, with the inverse association levelling off beyond approximately 2,050 units/day for MIMS-ES and 80 min/day for Time-ES. Among participants with sarcopenia, higher exercise snack levels were associated with a lower risk of all-cause mortality, with MIMS-ES (HR=0.83, 95% CI: 0.74, 0.91) and Time-ES (HR=0.80, 95% CI: 0.71, 0.91). The results were broadly consistent across subgroup and sensitivity analyses. These findings suggest that higher levels of exercise snacks were associated with favorable muscle health and survival outcomes, while further prospective and interventional studies are needed to clarify these associations.

## INTRODUCTION

Sarcopenia is a progressive systemic skeletal muscle disorder characterized by a gradual decline in both muscle mass and function [1, 2]. In recent years, it has been increasingly recognized as a major contributor to frailty, falls, functional impairment, and mortality [3, 4]. Epidemiological studies suggest that sarcopenia affects approximately 10% to 16% of older adults worldwide [5]. Beyond its impact on mobility and independence, sarcopenia substantially reduces quality of life, increases care dependency [6], and markedly elevates all-cause mortality, particularly in the presence of chronic comorbidities [7]. Consequently, identifying modifiable factors and practical approaches that may help prevent or delay sarcopenia has become an important public health and clinical priority.

Current evidence indicates that regular physical activity and structured exercise interventions, particularly resistance training and multicomponent exercise programs, are important components of the prevention and management of sarcopenia [8, 9]. The 2018 International Clinical Practice Guideline for Sarcopenia (ICFSR) identifies physical activity as a key component of sarcopenia management [10]. Furthermore, a recent randomized controlled trial of a multicomponent intervention demonstrated that moderate-intensity exercise was associated with a 22% lower incidence of mobility disability among individuals with frailty and sarcopenia [11]. Nevertheless, conventional exercise prescriptions often emphasize continuous sessions of at least 30 min at moderate-to-vigorous intensity [12], which may be challenging for individuals with functional decline or those lacking established exercise habits.

Recently, researchers have increasingly focused on short bouts of accumulated moderate-to-vigorous physical activity in daily life, commonly referred to as “exercise snacks” [13], although their operational definitions vary across studies. Exercise snacks may include both planned and structured exercise bouts and brief, intermittent movements embedded in everyday tasks (e.g., walking and stair climbing) [14]. Given their brief and flexible nature, exercise snacks may represent a potentially relevant way to accumulate physical activity for individuals who are unable or unwilling to engage in traditional continuous exercise [15]. Emerging evidence suggests that exercise snacks may be associated with a reduced risk of cognitive decline, a lower incidence of frailty, and improved metabolic health outcomes [16].

Although exercise snacks have attracted growing interest as an alternative way of accumulating physical activity, their associations with sarcopenia prevalence and long-term survival remain unclear. Therefore, we used accelerometer-derived indices of exercise snacking in a nationally representative sample from the 2011–2014 National Health and Nutrition Examination Survey (NHANES) to examine whether exercise snacks were associated with sarcopenia, handgrip strength, and all-cause mortality, thereby extending current evidence on the potential relevance of exercise snacks to muscle health and survival in older adults. We hypothesized that higher levels of exercise snacks would be associated with a lower prevalence of sarcopenia, greater handgrip strength, and lower all-cause mortality risk.

## MATERIALS AND METHODS

### Study design and population

NHANES is a large-scale program conducted by the National Center for Health Statistics (NCHS), a division of the Centers for Disease Control and Prevention (CDC), designed to evaluate the health and nutritional status of the U.S. population. The survey employs a complex, multistage, stratified, cluster probability sampling design and collects data through household interviews, standardized physical examinations, and laboratory assessments. The NCHS Research Ethics Review Board approved the survey protocol (NHANES 2011–2012: Protocol #2011–17; NHANES 2013–2014: continuation of Protocol #2011–17), and written informed consent was obtained from all participants.

For the present analysis, we used data from the 2011–2014 NHANES cycles. Participants were included if they: (1) were adults with valid sarcopenia-related assessment data; (2) had complete and valid physical activity data from the ActiGraph accelerometer; and (3) provided complete information for essential covariates. A total of 19,931 participants were initially identified. We excluded 10,362 individuals with missing sarcopenia-related data, 5,655 with incomplete physical activity data, and 945 with missing covariates. Ultimately, 2,969 participants remained for the final analysis (Figure 1).

**FIG. 1:**
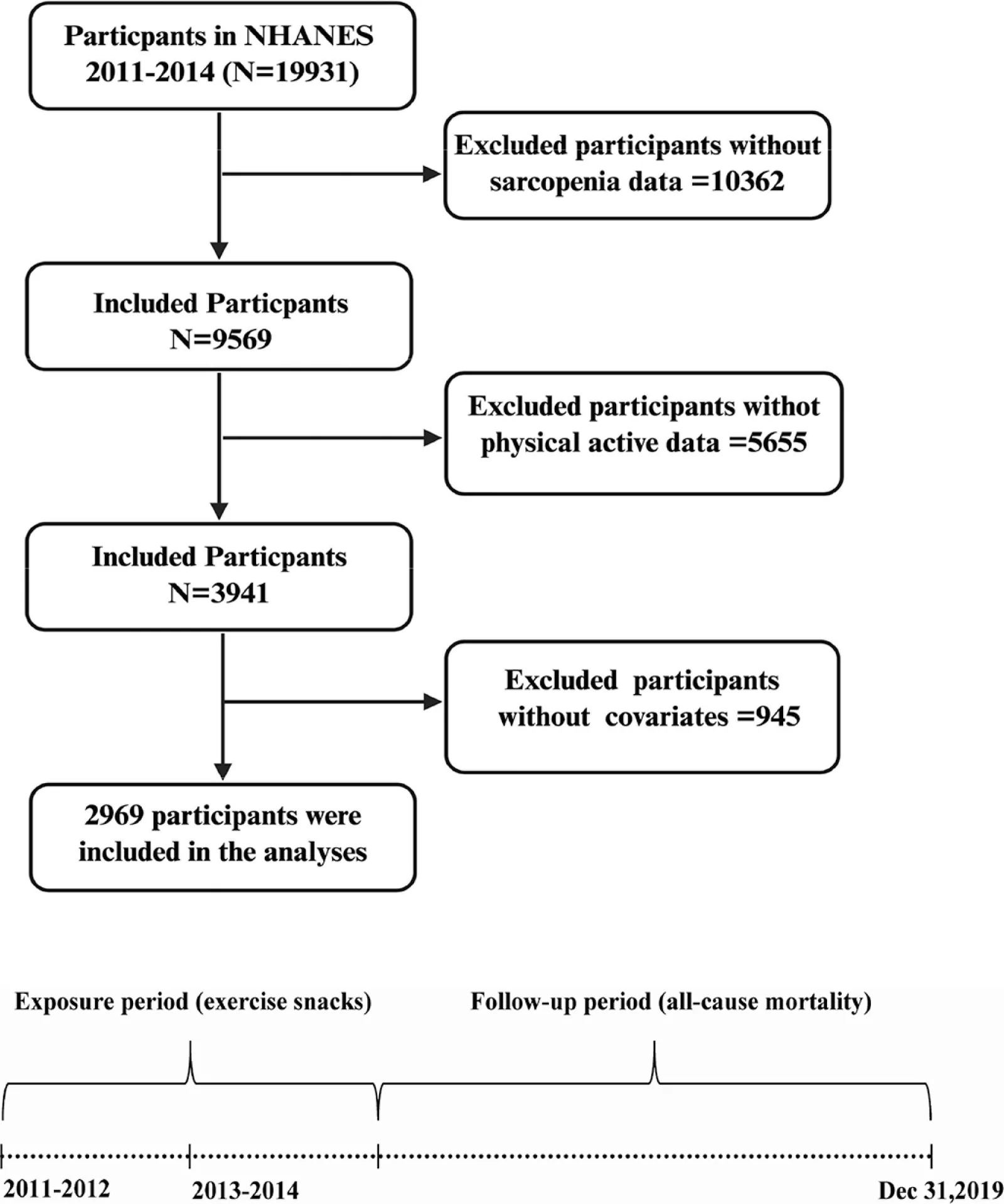
Flow chart of the screening of the NHANES 2011–2014 participants.

### Accelerometer and data processing

During the 2011–2014 NHANES cycles, participants were instructed to wear a wrist-worn triaxial accelerometer (ActiGraph GT3X+, Pensacola, FL, USA) continuously for 7 consecutive days to obtain objective measures of physical activity [17, 18]. Raw acceleration signals were recorded at 80 Hz and aggregated into one-minute epochs, which were subsequently converted into Monitor-Independent Movement Summary (MIMS) units as standardized metrics of activity [19]. Data from the first and last monitoring days were excluded because they did not represent complete 24-hour periods. A valid day was defined as having more than 16 hours of wear time, and participants were required to contribute at least 4 valid days to be included in the analysis [20].

Following prior studies, exercise snacks (ES) were defined as short bouts of moderate-to-vigorous physical activity lasting 2–5 minutes [21, 22]. Because there is currently no universally accepted accelerometer-based definition of ES, we adopted a 2–5-minute threshold as an operational definition, informed by previous literature and the 1-minute epoch structure of the available ActiGraph data. Accordingly, this threshold should be interpreted as a literature-informed operational definition rather than a validated accelerometer-based criterion. Data were processed using a customized R package to derive both volume (daily MIMS) and intensity measures [23]. Daily MIMS (units/day) was calculated as the sum of all MIMS/min values accumulated within a day, averaged across all valid wear days [24]. Intensity was determined by ranking individual MIMS/min values within each valid day, calculating the mean of the 30 highest values per day, and averaging these means across all valid days [19, 25]. Based on these definitions, two primary indicators were derived: (1) MIMS-ES (units/day), representing the average daily MIMS units accumulated during ES bouts; and (2) Time-ES (min/day), defined as the total daily minutes spent in ES across all qualifying bouts. Because MIMS-based classification of exercise snacks has not been specifically validated, MIMS-ES should be interpreted as an accelerometer-derived summary of movement accumulated during ES bouts rather than as a directly validated marker of exercise snack intensity. The use of MIMS for exercise snack classification and the operational definition of ES are provided in the Supplementary Methods.

### Definition of sarcopenia

Body composition was assessed using dual-energy X-ray absorptiometry (DXA). Appendicular skeletal muscle mass (ASM) was defined as the sum of lean soft tissue from all four limbs, and body mass index (BMI) was calculated from the measured height and weight. According to the criteria proposed by the Foundation for the National Institutes of Health (FNIH), sarcopenia was defined as an ASM/ BMI ratio of < 0.789 kg/m^2^ in men and < 0.512 kg/m^2^ in women [26]. All participants underwent standardized whole-body DXA scans at the Mobile Examination Center to ensure consistent data acquisition and uniform assessment of sarcopenia.

### Assessment of combined grip strength

Combined grip strength (kg) was assessed using a Takei Digital Grip Strength Dynamometer (Model TKK-5401; Takei Scientific Instruments, Niigata, Japan). The dynamometer was adjusted to fit each participant’s hand size, and the participants were instructed to exert maximal force. Each hand was tested thrice, with a 60-second rest interval between trials. Measurements were considered valid if the participants maintained the correct posture and the index finger formed a 90° angle with the dynamometer handle [27]. The combined grip strength was calculated as the sum of the highest recorded values from both hands.

### Ascertainment of mortality

All-cause mortality among participants was ascertained using publicly available NHANES-linked mortality files until December 31, 2019. The National Center for Health Statistics (NCHS) generated these files through probabilistic linkage with the National Death Index (NDI). The primary outcome was all-cause mortality, defined as death from any cause of death.

### Covariates

Based on previous research, key potential confounders were identified [28–30]. Demographic variables included age (years), sex (male or female), race (Mexican American, Other Hispanic, Non-Hispanic White, Non-Hispanic Black, and Other Race), education level (less than 9^th^ grade, 9–11^th^ grade, high school graduate, some college or AA degree, and college graduated or above), marital status (Married, Widowed, Divorced, Separated, Never married, and Living with partner), poverty-to-income ratio (PIR) categorized into three groups: < 1.30, 1.30–3.50, > 3.50; and body mass index (BMI, kg/m^2^). Smoking status was classified into three categories: never (< 100 cigarettes in lifetime), former (≥ 100 cigarettes in lifetime but not currently smoking), and current (≥ 100 cigarettes in lifetime and currently smoking). Alcohol consumption was categorized as never (< 12 drinks in lifetime), former (≥ 12 drinks in lifetime but no alcohol intake in the previous year), current light/moderate (≤ 1 drink/day), and current heavy (> 1 drink/day).

Diabetes was defined as a self-reported physician diagnosis of diabetes, current use of insulin or oral hypoglycemic agents, fasting plasma glucose level ≥ 7.0 mmol/L, 2-hour plasma glucose level ≥ 11.1 mmol/L during an oral glucose tolerance test, random plasma glucose level ≥ 11.1 mmol/L, or glycated hemoglobin A1c (HbA1c) level ≥ 6.5%. Hypertension was defined as a self-reported physician diagnosis of hypertension, an average systolic blood pressure of ≥ 140 mmHg, and/or an average diastolic blood pressure of ≥ 90 mmHg, based on three consecutive measurements, or current use of antihypertensive medications. To further account for nutritional status and systemic inflammation, we additionally adjusted for serum albumin and dietary protein intake as nutritional indicators. White blood cell count (WBC) and systemic immune-inflammation index (SII) were included as inflammation markers. SII was computed as (*platelet count × neutrophil count) / lymphocyte count* based on complete blood count measurements. Dietary protein intake was derived from the 24-hour dietary recall data in NHANES.

### Statistical analysis

Given the complex, multistage, and stratified probability sampling design of the NHANES, all statistical analyses incorporated sampling weights (divided by two for combined cycles), primary sampling units (PSUs), and strata to generate nationally representative estimates. Continuous variables are expressed as mean ± standard deviation (SD), whereas categorical variables are presented as frequencies (n) and percentages (%). Differences in categorical variables were assessed using the chi-square test, and differences in continuous variables were evaluated using the t-test or Wilcoxon rank-sum test.

Survey-weighted multivariable logistic regression models were used to evaluate the association between exercise snacks (MIMS, MIMSES, and Time-ES) and sarcopenia. The exercise snack variables were categorized into quartiles (Q1–Q4). Odds ratios (ORs) and 95% confidence intervals (CIs) were calculated. Model I was unadjusted; Model II was adjusted for age, sex, race, marital status, educational level, and PIR; and Model III was further adjusted for smoking status, alcohol consumption, hypertension, diabetes, albumin, dietary protein intake, WBC, and SII. Survey-weighted multivariable linear regression models were used to examine the association between exercise snacks and combined grip strength. Prior to fitting the final fully adjusted models, we assessed multicollinearity among covariates using variance inflation factors (VIFs); VIF values ≥ 5 were considered indicative of potentially problematic multicollinearity.

During the follow-up period, among participants with sarcopenia, we calculated incidence rates across exercise snack quartiles and fitted multivariable Cox proportional hazards regression models to examine the associations between exercise snacks and all-cause mortality, including three progressively adjusted models to account for potential confounders. Results were reported as hazard ratios (HRs) and 95% confidence intervals (CIs). In addition, all-cause mortality outcomes according to exercise snack levels were assessed using Kaplan–Meier (KM) survival curves and log-rank tests. To explore the potential nonlinear associations between exercise snacks, sarcopenia, and all-cause mortality, restricted cubic spline (RCS) models were constructed after adjusting for potential confounders.

Sensitivity analyses included subgroup analyses, multiple imputation (MICE), propensity score matching (PSM) and lagged analyses to evaluate the robustness of the main findings. Based on prior evidence [31, 32] suggesting that demographic and lifestyle factors may influence muscle mass and mortality, stratified analyses were conducted by age, sex, race, educational level, marital status, PIR, BMI, smoking status, alcohol consumption, diabetes, and hypertension. Within each subgroup, we fitted the same fully adjusted model as in the main analysis, adjusting for the same covariates except the stratification variable. The primary analyses were conducted using a complete-case approach. To assess the robustness of the findings to missing covariate data, we additionally performed MICE as a sensitivity analysis under the assumption that data were missing at random (MAR). Given the substantial reduction from the initial NHANES sample to the final complete-case analytic sample, this sensitivity analysis was also used to evaluate the stability of the main findings under an alternative missing-data handling approach. PSM was conducted to balance baseline characteristics between participants with and without sarcopenia using 1:1 matching. To reduce the potential for reverse causation, we performed lagged sensitivity analyses by excluding participants who died within the first two years of follow-up and re-estimating survey-weighted Cox proportional hazards models. Detailed descriptions are provided in Supplementary Methods. All analyses were performed using R software (version 4.4.1, R Foundation of Statistical Computing, Vienna, Austria), with statistical significance defined as *P* < 0.05.

## RESULTS

### Baseline characteristics

A total of 2,969 participants were included in the present study, with a mean age of 66.61 years; 1,495 (51%) were men and 1,474 (49%) were women. Among them, 459 participants were identified as having sarcopenia. Significant differences were observed between participants with and without sarcopenia in terms of age, race, educational level, marital status, PIR, BMI, smoking and drinking status, and the prevalence of hypertension and diabetes mellitus. Compared with non-sarcopenic individuals, those with sarcopenia exhibited lower combined grip strength, albumin levels, protein intake, total MIMS, MIMS-ES, and Time-ES levels. The detailed baseline characteristics are presented in Table 1.

**TABLE 1:** Baseline characteristics of participants with and without sarcopenia

Characteristic	Overall N = 2969	Non-Sarcopenia N = 2510	Sarcopenia N = 459	*P*-Value
**Age (years)**	66.61 ± 7.71	64.74 ± 7.29	68.86 ± 9.84	**< 0.001**

**Sex, n (%)**				0.5
Male	1495 (51%)	1262 (51%)	233 (53%)	
Female	1474 (49%)	1248 (49%)	226 (47%)	

**Race, n (%)**				**< 0.001**
Mexican American	381 (8.7%)	282 (7.5%)	99 (16%)	
Other Hispanic	271 (5.7%)	223 (5.4%)	48 (7.3%)	
Non-Hispanic White	1204 (68%)	1049 (69%)	155 (60%)	
Non-Hispanic Black	624 (10%)	546 (11%)	78 (8.8%)	
Other Race	489 (7.8%)	410 (7.8%)	79 (8.1%)	

**Education, n (%)**				**0.018**
Less than 9^th^ grade	170 (3.7%)	129 (3.2%)	41 (6.5%)	
9–11^th^ grade	382 (10%)	313 (9.9%)	69 (11%)	
High school graduate	643 (21%)	546 (22%)	97 (21%)	
Some college or AA degree	895 (30%)	751 (30%)	144 (32%)	
College graduate or above	879 (34%)	771 (35%)	108 (29%)	

**Marital, n (%)**				**0.007**
Married	1762 (63%)	1505 (64%)	257 (55%)	
Widowed	57 (1.6%)	50 (1.6%)	7 (1.9%)	
Divorced	418 (14%)	359 (14%)	59 (15%)	
Separated	129 (3.1%)	106 (2.9%)	23 (4.1%)	
Never married	399 (12%)	319 (11%)	80 (17%)	
Living with partner	204 (6.0%)	171 (5.7%)	33 (7.4%)	

**PIR, n (%)**				**0.003**
< 1.3	893 (19.90%)	730 (19.06%)	163 (25.00%)	
1.3–3.5	955 (32.08%)	799 (31.41%)	156 (36.13%)	
> 3.5	1121 (48.02%)	981 (49.54%)	140 (38.87%)	

**BMI (Kg/m^2^)**	29.13 ± 5.87	28.81 ± 5.75	31.02 ± 6.22	**< 0.001**

**Smoking, n (%)**				**0.010**
Never	1679 (57%)	1391 (54%)	288 (62%)	
Former	589 (20%)	509 (23%)	80 (17%)	
Current	701 (23%)	610 (23%)	91 (22%)	

**Drinking, n (%)**				**0.001**
Never	696 (23.4%)	547 (21.8%)	149 (32.5%)	
Former	271 (9.1%)	226 (9.0%)	45 (9.8%)	
Light/Moderate	893 (30.1%)	775 (30.9%)	118 (25.7%)	
Heavy	1109 (37.4%)	962 (38.3%)	147 (32.0%)	

**Diabetes, n (%)**				**0.030**
Yes	317 (8%)	265 (8%)	52 (10%)	
No	2652 (92%)	2245 (92%)	407 (90%)	

**Hypertension, n (%)**				**0.017**
Yes	920 (30%)	771 (30%)	149 (29%)	
No	2049 (70%)	1739 (70%)	310 (71%)	

**Albumin (g/L)**	42.91 ± 3.09	42.99 ± 3.06	42.46 ± 3.20	**0.025**

**Intake of protein (gm/d)**	70.48 ± 4.46	70.69 ± 4.42	70.45 ± 4.47	**0.04**

**WBC (X10** ^9^ **/L)**	7.21 ± 2.19	7.17 ± 2.19	7.50 ± 2.22	**0.014**

**SII**	525.31 ± 279.49	521.72 ± 279.88	547.71 ± 276.32	**0.019**

**Combined grip strength (kg)**	54.5 ± 18.3	58.1 ± 18.7	51.1 ± 18.2	**0.017**

**MIMS (unit/day)**	5045.81 ± 1960.66	5117.52 ± 1975.06	4613.13 ± 1814.24	**< 0.001**

**MIMS-ES (unit/day)**	2013.16 ± 778.73	2046.04 ± 774.34	1814.78 ± 776.52	**0.003**

**Time-ES (min/day)**	85.43 ± 32.66	90.28 ± 30.20	76.19 ± 31.73	**< 0.001**

mean ± standard deviation (SD) for continuous; n (%) for categorical; PIR, poverty income ratio; BMI, body mass index; WBC, White blood cell count; SII, systemic immune-inflammation index; MIMS, Monitor-Independent Movement Summary; MIMS-ES, daily average MIMS specific to exercise snacks; Time-ES, daily average time spent on exercise snacks.

### Association of exercise snacks with sarcopenia

VIF diagnostics indicated no problematic multicollinearity among covariates (range: 1.01 to 1.28, Supplementary Fig. S1). Across all three logistic regression models (Table 2), we found that exercise snacks were associated with lower odds of sarcopenia. In the fully adjusted model (Model III), each additional unit per day of MIMS-ES was associated with an 8% reduction in the odds of sarcopenia (OR = 0.92, 95% CI: 0.88, 0.96). When the exposure was expressed as time spent on exercise snacks, each additional minute per day of Time-ES corresponded to a 9% reduction in the odds of sarcopenia (OR = 0.91, 95% CI: 0.88, 0.96). Quartile-based analyses further confirmed these associations, with all trend tests reaching statistical significance (all *P* for trend < 0.05), indicating that participants in the highest quartile had lower odds of sarcopenia than those in the lowest quartile.

**TABLE 2:** Association between exercise snacks and sarcopenia analyzed using logistic regression

	Model I		Model II		Model III	

	OR (95% CI)	*P* value	OR (95% CI)	*P* value	OR (95% CI)	*P* value
**MIMS (unit/day)**	0.97 (0.93, 0.99)	0.003	0.94 (0.88, 0.97)	0.004	0.92 (0.86, 0.95)	0.001
Q1	1.0		1.0		1.0	
Q2	0.75 (0.55, 0.91)	0.006	0.72 (0.58, 0.88)	0.007	0.77 (0.63, 0.94)	0.031
Q3	0.65 (0.48, 0.90)	0.005	0.62 (0.47, 0.86)	0.005	0.62 (0.45, 0.84)	0.012
Q4	0.57 (0.39, 0.78)	0.001	0.56 (0.39, 0.80)	0.004	0.55 (0.39, 0.83)	0.009
***P* for trend**	< 0.001		0.002		0.006	

**MIMS-ES (unit/day)**	0.96 (0.92, 0.98)	< 0.001	0.95 (0.91, 0.98)	< 0.001	0.92 (0.88, 0.96)	< 0.001
Q1	1.0		1.0		1.0	
Q2	0.66 (0.45, 0.78)	0.034	0.63 (0.42, 0.84)	0.028	0.62 (0.40, 0.95)	0.032
Q3	0.58 (0.41, 0.74)	0.005	0.57 (0.39, 0.83)	0.007	0.53 (0.36, 0.77)	0.005
Q4	0.40 (0.25, 0.64)	< 0.001	0.37 (0.22, 0.62)	0.001	0.34 (0.21, 0.56)	0.001
***P* for trend**	< 0.001		0.002		0.002	

**Time-ES (min/day)**	0.96 (0.92, 0.99)	< 0.001	0.95 (0.92, 0.97)	< 0.001	0.91 (0.88,0.96)	< 0.001
Q1	1.0		1.0		1.0	
Q2	0.66 (0.41, 0.83)	0.022	0.65 (0.43, 0.82)	0.020	0.62 (0.38, 0.78)	0.013
Q3	0.56 (0.35, 0.80)	0.015	0.50 (0.28, 0.79)	0.005	0.47 (0.30, 0.74)	0.002
Q4	0.44 (0.26, 0.64)	0.004	0.40 (0.25, 0.62)	< 0.001	0.38 (0.20, 0.59)	< 0.001
***P* for trend**	< 0.001		0.001		0.002	

CI, Confidence Interval; MIMS, Monitor-Independent Movement Summary; MIMS-ES, daily average MIMS specific to exercise snacks; Time-ES, daily average time spent on exercise snacks.

Model I was unadjusted for covariates;

Model II was adjusted for age, sex, race, marital status, educational level and poverty income ratio (PIR);

Model III was adjusted for age, sex, race, marital status, educational level and PIR, smoking status, alcohol consumption, hypertension, diabetes, albumin, intake of protein, WBC and SII.

The RCS models further characterized the association between exercise snacks and sarcopenia. As shown in Figure 2, the association between total MIMS and sarcopenia was approximately linear (P-non-linear = 0.2442), with the odds of sarcopenia decreasing as MIMS increased above the reference value (Reference = 4890 units/ day). In contrast, both MIMS-ES and Time-ES showed significant nonlinear relationships with sarcopenia (MIMS-ES: P-non-linear = 0.0004; Time-ES: P-non-linear < 0.001): the odds of sarcopenia declined more steeply at lower exposure levels, and the curves flattened around the reference levels (MIMS-ES = 2050 units/day; Time-ES = 80 total min/day), indicating a plateauing association at higher exposure levels.

**FIG. 2:**
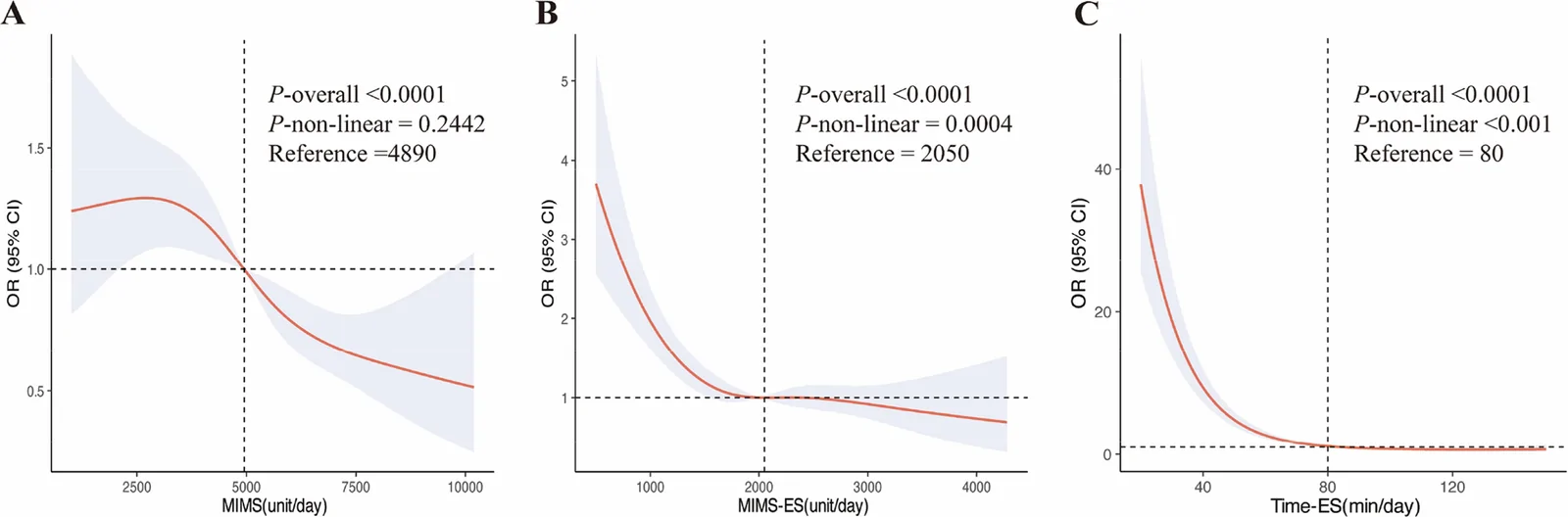
Association between exercise snacks and sarcopenia in NHANES 2011–2014 participants by RCS. MIMS, Monitor-Independent Movement Summary; MIMS-ES, daily average MIMS specific to exercise snacks; Time-ES, daily average time spent on exercise snacks; NHANES, National Health and Nutrition Examination Survey; RCS, restricted cubic spline; CI, Confidence Interval.

### Association between exercise snacks and combined grip strength

Linear regression analysis showed a positive association between exercise snacks and combined grip strength (Table 3). In the unadjusted Model I, increases in total MIMS (β = 0.01, 95% CI: 0.0032, 0.075, *P* = 0.02), MIMS-ES (β = 0.01, 95% CI: 0.0022, 0.13, *P* = 0.02), and Time-ES (β = 0.03, 95% CI: 0.0011, 0.14, *P* = 0.028) were all positively associated with higher grip strength. These associations remained statistically significant in the fully adjusted model (Model III). Specifically, each unit increase in MIMS was associated with a 0.03 kg increase in grip strength (95% CI: 0.0027, 0.19, *P* = 0.007), whereas each unit increase in MIMS-ES corresponded to a 0.04 kg increase (95% CI: 0.0015, 0.28, *P* = 0.006). Moreover, every additional minute per day of Time-ES was significantly associated with a 0.08 kg increase in grip strength (95% CI: 0.0033, 0.31, *P* = 0.001).

**TABLE 3:** The association between exercise snacks and combined grip strength.

	Model I		Model II		Model III	

	β (95% CI)	*P* value	β (95% CI)	*P* value	β (95% CI)	*P* value
MIMS (unit/day)	0.01 (0.0032, 0.07)	0.020	0.01 (0.009, 0.10)	0.030	0.03 (0.0027, 0.19)	0.007
MIMS-ES (unit/day)	0.01 (0.0022, 0.13)	0.020	0.03 (0.0040, 0.19)	0.016	0.04 (0.0015, 0.28)	0.006
Time-ES (min/day)	0.03 (0.0011, 0.14)	0.028	0.04 (0.0010, 0.17)	0.006	0.08 (0.0033, 0.31)	0.001

CI, Confidence Interval; MIMS, Monitor-Independent Movement Summary; MIMS-ES, daily average MIMS specific to exercise snacks; Time-ES, daily average time spent on exercise snacks.

Model I was unadjusted for covariates;

Model II was adjusted for age, sex, race, marital status, educational level and poverty income ratio (PIR);

Model III was adjusted for age, sex, race, marital status, educational level and PIR, smoking status, alcohol consumption, hypertension, diabetes, albumin, intake of protein, WBC and SII.

### Exercise snacks, sarcopenia, and all-cause mortality

During a median follow-up of 6.8 years, 261 deaths were documented among the 2,969 participants (Supplementary Table S1). KM survival curves stratified by sarcopenia status (Supplementary Fig. S2) showed that participants with sarcopenia had significantly lower survival probabilities throughout the follow-up period compared with those without sarcopenia. The two curves separated early and remained apart over time, with the sarcopenia group exhibiting a higher cumulative mortality rate at the end of follow-up. The log-rank test revealed a highly significant difference between the groups (*P* < 0.0001).

Figure 3 depicts the associations between exercise snacks (MIMS, MIMS-ES, and Time-ES) and all-cause mortality after multivariable adjustment. For MIMS-ES, higher activity levels were inversely associated with all-cause mortality in the fully adjusted model (HR = 0.83, 95% CI: 0.74, 0.91). Similarly, in the analysis of Time-ES, each additional minute per day was associated with a lower hazard of all-cause mortality (HR = 0.80, 95% CI: 0.71, 0.91). Quartile-based analyses further showed that participants in the highest quartile (Q4) had a lower hazard of all-cause mortality than those in the lowest quartile (Q1) (*P* for trend < 0.05). To further examine potential nonlinear associations, RCS models were applied. After adjusting for covariates, nonlinear relationships were observed between MIMS, MIMS-ES, Time-ES, and all-cause mortality (*P*-nonlinear < 0.001), as shown in Supplementary Fig. S3.

**FIG. 3:**
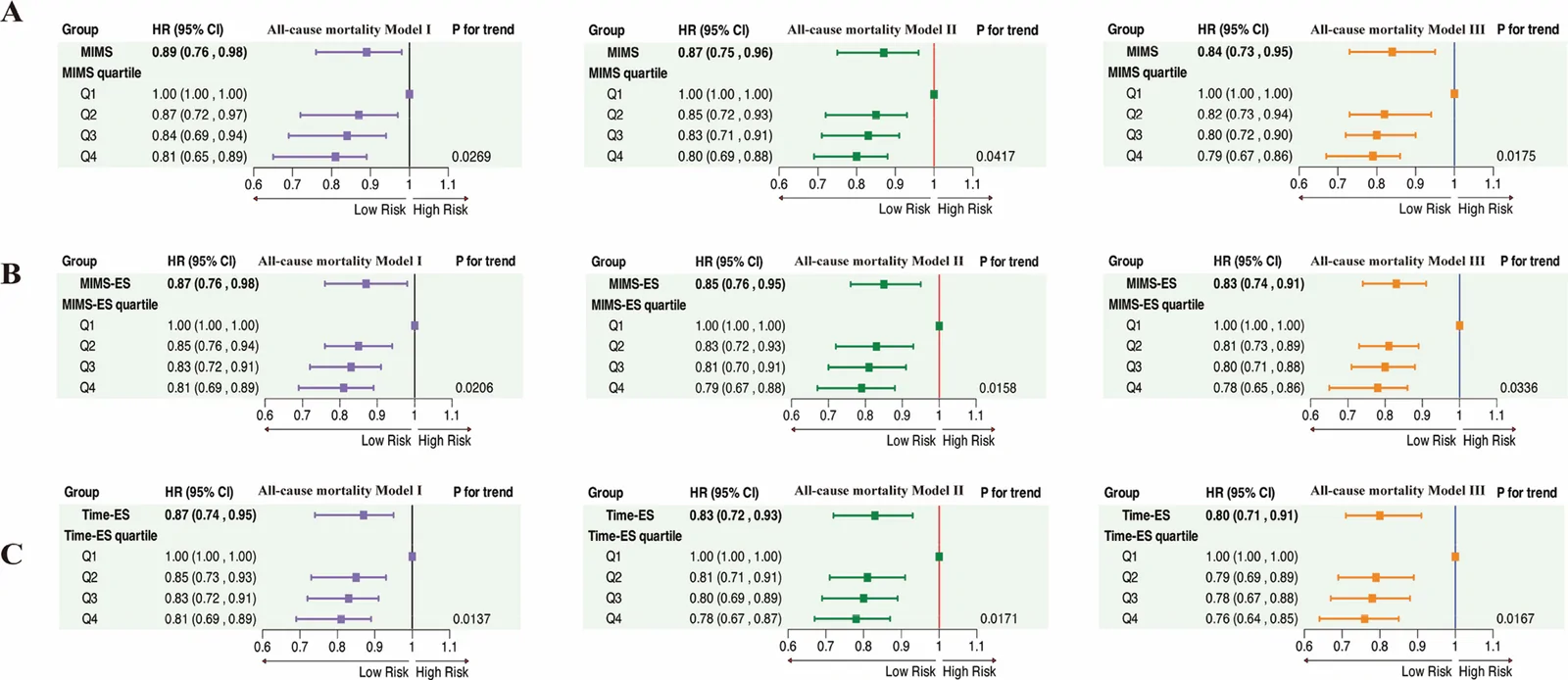
Association of exercise snacks with all-cause mortality in participants from the NHANES 2011–2014. MIMS, Monitor-Independent Movement Summary; MIMS-ES, daily average MIMS specific to exercise snacks; Time-ES, daily average time spent on exercise snacks; HR, Hazard ratios Model I was unadjusted for covariates. Model II was adjusted for age, sex, race, marital status, education level, and poverty income ratio (PIR). Model III was adjusted for age, sex, race, marital status, educational level and PIR, smoking status, alcohol consumption, hypertension, diabetes, combined grip strength, albumin, intake of protein, WBC and SII.

The KM survival curves also demonstrated separation in all-cause mortality across the exercise snack quartiles. As shown in Figure 4A, participants in the highest MIMS-ES quartile (Q4) exhibited significantly higher survival probabilities than those in the lowest quartile (Q1), with an early divergence in survival curves that persisted over time (log-rank test, *P* < 0.0001). A similar pattern was observed for Time-ES (Figure 4B), in which individuals with greater accumulated daily exercise snack time showed lower mortality than those with minimal engagement. The stepwise separation of curves across quartiles suggests that higher levels of exercise snacks were associated with better survival outcomes.

**FIG. 4:**
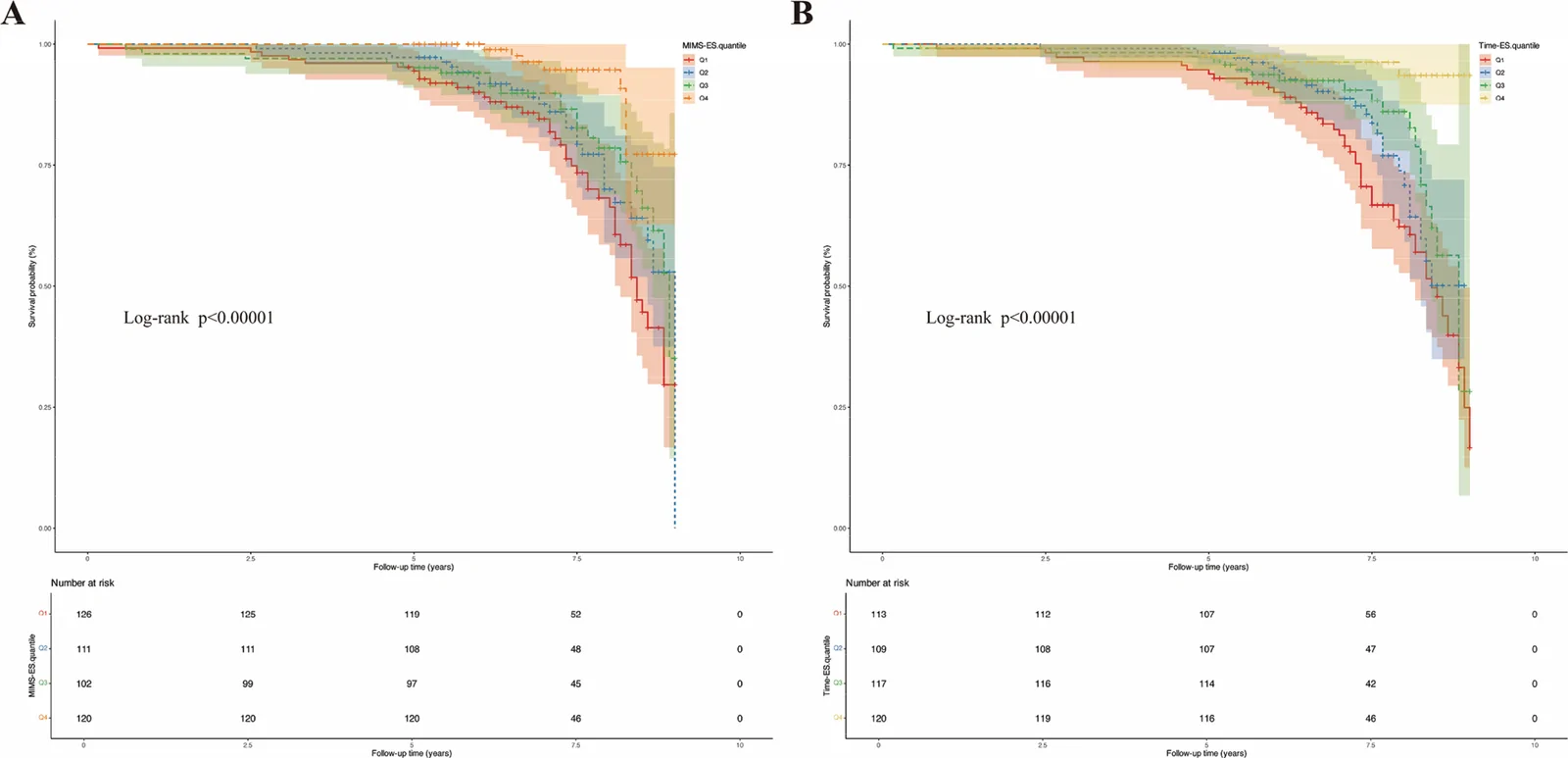
Kaplan–Meier survival curves for all-cause mortality according to exercise snack levels among participants with sarcopenia. (A) Survival curves stratified by quartiles of MIMS-ES (daily mean Monitor-Independent Movement Summary units accumulated during exercise snacks). (B) Survival curves stratified by quartiles of Time-ES (daily minutes spent in exercise snacks)

### Sensitivity analyses

Forest plots were generated to depict the association between exercise snacks (MIMS, MIMS-ES, and Time-ES) and sarcopenia and all-cause mortality. Stratified analyses were conducted according to age, sex, race, educational level, marital status, PIR, BMI, smoking and alcohol status, diabetes, and hypertension (Fig. 5). Subgroup analyses and interaction tests demonstrated that the associations between exercise snacks and all-cause mortality were consistent across all subgroups after multivariable adjustment, with no significant interactions observed (*P* for interaction > 0.05). The results of the sensitivity analyses were consistent with those obtained from the primary analysis, supporting the robustness of the main findings (Supplementary Tables 2–8).

**FIG. 5:**
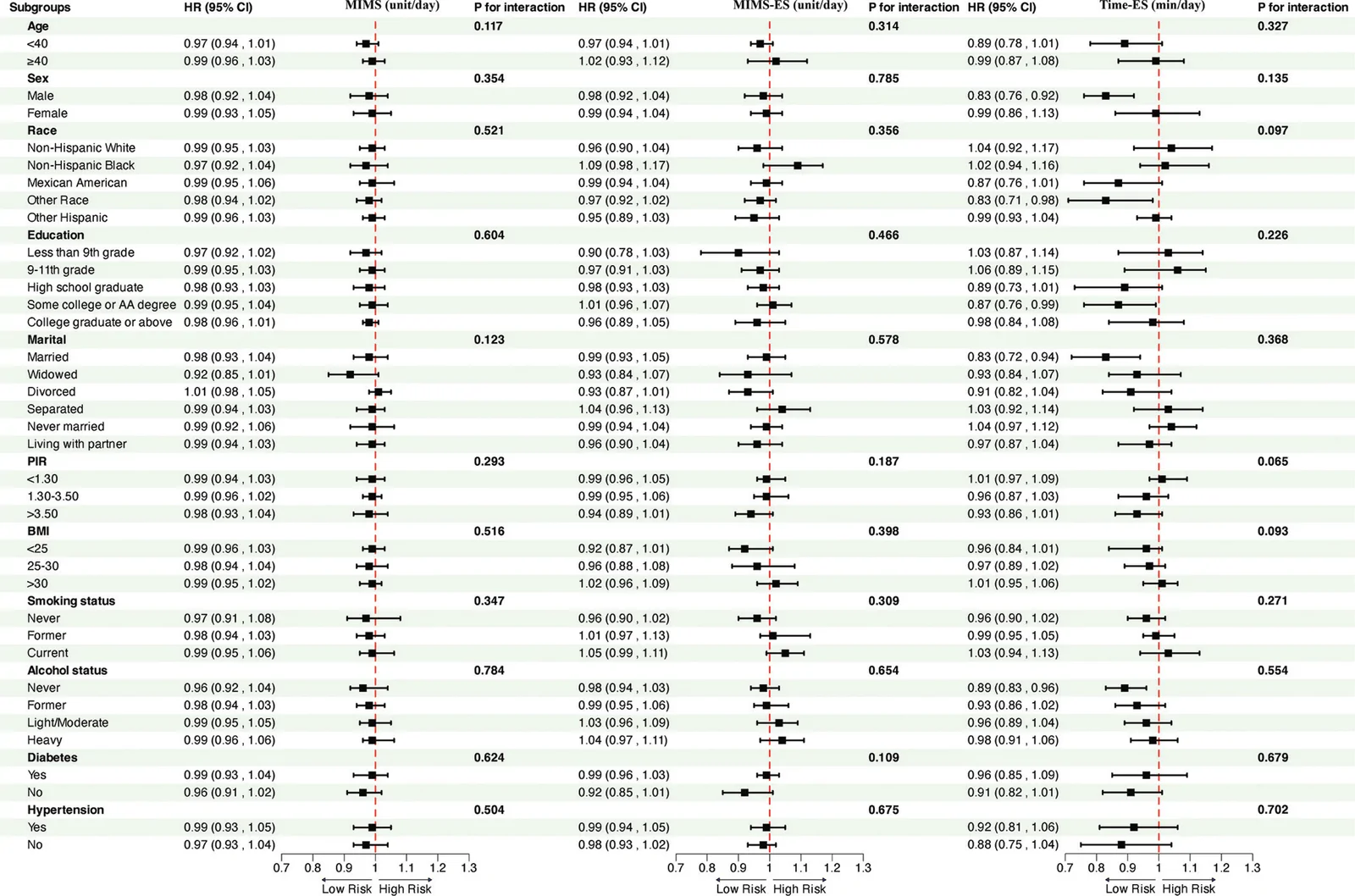
Subgroup analysis of the association between exercise snacks and all-cause mortality. CI: Confidence Interval; PIR: poverty income ratio, BMI: body mass index; MIMS, Monitor-Independent Movement Summary; MIMS-ES, daily average MIMS specific to exercise snacks; Time-ES, daily average time spent on exercise snacks.

## DISCUSSION

Sarcopenia is a major contributor to functional impairment and mortality in older adults [4, 33]. Although an expanding body of evidence suggests that physical activity is associated with favorable sarcopenia-related health outcomes, evidence regarding exercise snacks remains relatively limited, and their operational definitions are not yet fully standardized across studies [34, 35]. To our knowledge, few population-based studies have examined the association of exercise snacks with sarcopenia and all-cause mortality. Our findings indicate that higher levels of exercise snacks were associated with a lower prevalence of sarcopenia, greater handgrip strength, and lower all-cause mortality. These associations remained generally consistent across multiple model adjustments and subgroup analyses, supporting the robustness of our results.

As brief, fragmented bouts of moderate-to-vigorous physical activity embedded in daily life, exercise snacks have emerged as a potentially relevant pattern of physical activity accumulation that may confer health benefits similar to those observed in some studies of more traditional exercise patterns, while potentially reducing reliance on prolonged continuous activity [13, 36]. A large-scale multicenter retrospective cohort study reported that higher physical activity levels were significantly associated with a reduced risk of fibrosis, sarcopenia, and atherosclerotic cardiovascular disease [37]. Our study showed that exercise snacks were associated with a lower prevalence of sarcopenia. Compared with previous studies, our findings further suggest that the associations of MIMS-ES and Time-ES with sarcopenia may not be strictly linear. Differences across studies may be related to variations in the definition of exercise snacks, physical activity assessment methods, participant characteristics, and outcome ascertainment [38]. The inverse associations of exercise snacks with sarcopenia became less pronounced beyond approximately 2050 units/day for MIMS-ES or 80 min/day for Time-ES. This pattern suggests that accumulated short bouts of daily activity may represent a relevant form of physical activity accumulation, although the association may not strengthen uniformly across the full exposure range.

Handgrip strength is widely recognized as a key indicator of sarcopenia and has been recommended by both the EWGSOP2 and AWGS guidelines as a reliable measure of muscle function [39, 40]. Beyond its diagnostic value, previous studies have shown that low handgrip strength is significantly associated with an increased risk of falls, functional impairment, cardiovascular events, and all-cause mortality [41, 42]. In our study, we observed a positive association between higher levels of exercise snacks and greater combined hand-grip strength, with each additional minute per day of Time-ES corresponding to a 0.08 kg increase in grip strength [43]. This association may partly explain the observed inverse association between exercise snacks and sarcopenia. Supporting our findings, a cross-sectional study reported that engaging in sufficient aerobic and resistance exercise was associated with higher handgrip strength, which may help explain the association between exercise snacks and lower sarcopenia prevalence observed in our study.

An expanding body of evidence suggests that sarcopenia is an independent predictor of frailty and all-cause mortality [3, 7]. Multiple longitudinal studies and systematic reviews have reported that individuals with sarcopenia face a higher risk of all-cause and cause-specific mortality compared with their non-sarcopenic counter-parts [44, 45]. Our study further examined the relationship between exercise snacks and mortality risk in individuals with sarcopenia. We found that higher levels of exercise snacks (MIMS-ES and Time-ES) were significantly associated with a lower risk of all-cause mortality in participants with sarcopenia, with an inverse association observed across exposure levels. In addition, we conducted a lagged sensitivity analysis, and the associations between exercise snacks and mortality remained statistically significant, which supports the robustness of these findings. In recent years, the inverse association between physical activity and mortality has been reported in diverse populations [46, 47]. Notably, our study adds to this evidence by indicating that although sarcopenia increases the risk of premature mortality, higher levels of brief, intermittent moderate-to-vigorous physical activity embedded in daily life were associated with lower mortality risk. Furthermore, recent literature suggests that the pattern in which physical activity is accumulated may also be relevant to health outcomes [48]. In particular, emerging evidence indicates that fragmented or intermittently accumulated activity patterns are associated with mortality-related outcomes [49].

Although our findings suggest that exercise snacks were associated with greater handgrip strength and lower risks of sarcopenia and mortality, the underlying mechanisms remain unclear. Based on the current evidence, several plausible pathways can be proposed. Brief bouts of moderate-to-vigorous physical activity may help stimulate muscle protein synthesis, improve mitochondrial function, and enhance insulin sensitivity, which may together contribute to the preservation of muscle mass and function [50–52]. In addition, accumulating brief bouts of moderate-to-vigorous activity across the day may also be relevant to cardiorespiratory and cardiometabolic health. Specifically, these activity patterns may elicit short-term cardiovascular and metabolic responses that, when accumulated over time, could contribute to cardiorespiratory adaptation without requiring prolonged continuous exercise [17]. Recent reviews have suggested that exercise snacks may improve cardiorespiratory fitness and may favorably influence selected cardiometabolic markers, such as postprandial glucose regulation and blood pressure [36]. From a behavioral perspective, prolonged sedentary time has been associated with adverse cardiometabolic and mortality outcomes [53, 54]. In this context, exercise snack short duration and flexibility may help interrupt sedentary time and promote the accumulation of daily physical activity, which may be relevant to longer-term adherence and overall health [55].

Our study has several strengths as well as limitations. Its strengths include the use of a large, nationally representative sample, accelerometer-based objective assessment of physical activity, and the simultaneous examination of sarcopenia, handgrip strength, and all-cause mortality. These features provide population-based evidence on the potential relevance of fragmented physical activity patterns to muscle health and survival in older adults. However, several limitations of this study should be acknowledged. First, the cross-sectional and observational design precludes causal inference. Second, the 2–5-minute threshold used to define exercise snacks was a literature-informed operational definition rather than a validated accelerometer-based standard, and MIMS-ES should be interpreted as an accelerometer-derived summary of movement accumulated during these bouts rather than as a directly validated marker of exercise snack intensity. Accordingly, some degree of misclassification in the identification of exercise snack behavior is possible. Third, although we adjusted for a broad range of covariates, residual confounding from unmeasured or imperfectly measured factors cannot be excluded. In addition, given the number of models and subgroup analyses performed, the possibility of chance findings should also be considered. The consistency of the results across sensitivity analyses and multiple imputation provides some reassurance regarding the stability of the observed associations. Finally, the substantial reduction from the initial NHANES sample to the final complete-case analytic sample may have introduced selection bias and reduced representativeness. Therefore, caution is warranted when generalizing these findings to the broader NHANES population, although the results were broadly consistent in multiple imputation analyses.

## Supplementary Material

Associations of exercise snacks with sarcopenia and all-cause mortality: A national cohort study

## Data Availability

For a comprehensive description of the NHANES survey methodology and data sources, please visit the website (https://wwwn.cdc.gov/nchs/nhanes/default.aspx). All relevant data supporting the findings of this study are included in the article and its supplementary material.

## CONCLUSIONS

In conclusion, our study found that higher levels of exercise snacks were associated with a lower prevalence of sarcopenia, greater hand-grip strength, and a lower risk of all-cause mortality among older adults. These findings suggest that brief, accumulated bouts of moderate-to-vigorous physical activity performed throughout daily life may be relevant to muscle health and survival. Given the observational design of this study, further prospective and interventional studies are needed to clarify these associations.
