# Supplementary material for: Associations of exercise snacks with sarcopenia and all-cause mortality: A national cohort study
Source: Biol Sport. 2026 Jul 27;43:1615–39. doi: 10.5114/biolsport.2026.163369 (PMC13711647; doi:10.5114/biolsport.2026.163369)
Supplement: Supplementary file 1 [file JBS-43-58396-s1.pdf]

SUPPLEMENTARY MATERIALS

SUPPLEMENTARY METHODS

Accelerometer data processing

The raw triaxial acceleration data collected from the ActiGraph GT3X+ devices during the NHANES 2011–2014 cycles were processed using the Monitor-Independent Movement Summary (MIMS) algorithm [1]. MIMS is a standardized, device-independent summary metric developed to facilitate comparisons across wrist-worn accelerometer devices and processing platforms. The conversion of raw acceleration data into MIMS-units involves a four-step signal processing procedure, including interpolation, extrapolation, bandpass filtering, and axis-specific aggregation [2]. This processing framework is intended to preserve informative movement signals while reducing noise and device-related artifacts. Accordingly, MIMS-units provide an objective summary measure of accelerometer-derived movement that has been used in population-based analyses of wrist accelerometer data.

Because the main accelerometer processing steps and analytic inclusion criteria are described in the main Methods section, they are not repeated here in detail. In the present study, MIMS-based metrics were used to characterize both overall movement volume and movement accumulated during exercise snack bouts. Although MIMS is a standardized summary metric, its application to exercise snack–specific classification has not been specifically validated. Therefore, MIMS-ES should be interpreted as an accelerometer-derived summary of movement accumulated during ES bouts rather than as a directly validated marker of exercise snack intensity.

Derivation of exercise snack threshold

Exercise snacks (ES) were operationally defined as brief bouts of moderate-to-vigorous physical activity lasting 2–5 minutes. This definition was informed by previous literature and by the temporal resolution of the accelerometer-derived data [3, 4]. The NHANES physical activity monitor datasets processed via the MIMS algorithm are summarized at the minute level using a 1-minute epoch structure. Within this framework, activity bouts shorter than 2 minutes may be more difficult to classify consistently, particularly when distinguishing sustained moderate-to-vigorous movement from brief or sporadic arm motion [5, 6]. For this reason, a minimum threshold of 2 minutes was selected as an operational approach to improve the consistency of identifying sustained activity bouts within the available data. The upper limit of 5 minutes was applied to distinguish these brief, fragmented activity bouts from longer, more continuous exercise sessions, which are often defined differently in the broader physical activity literature [7]. We acknowledge that alternative definitions are possible and that shorter bouts may also be physiologically relevant; however, the present definition was selected to provide a consistent and reproducible framework for identifying fragmented activity bouts within the NHANES accelerometer dataset.

Based on this definition, two primary ES indicators were derived. First, MIMS-ES (units/day) was defined as the average daily MIMS units accumulated during ES bouts, representing the activity volume specifically accrued within exercise snack episodes. Second, Time-ES (min/day) was defined as the total daily minutes spent in ES across all qualifying bouts. For comparison, Total MIMS represented the overall daily movement volume accumulated across the full day, regardless of whether activity occurred within ES bouts [8, 9]. These measures were intended to capture complementary dimensions of fragmented physical activity: MIMS-ES reflects movement volume accrued during ES bouts, whereas Time-ES reflects the accumulated duration of ES participation.

Weighted analysis

Weighted analysis is a statistical method that assigns specific weights to different observations within a sample to reflect their varying importance and representativeness within the overall dataset [10, 11]. This approach effectively corrects biases inherent in sample estimations, thereby enhancing the precision and validity of statistical estimates. In the present study, we employed a weighted analysis approach by incorporating the complex sampling design and mobile examination center (MEC) sample weights recommended by NHANES guidelines, ensuring that our sample accurately represents the extensive diversity and generalizability of the U.S. adult population [12]. Consequently, this method significantly enhances the reliability and external validity of our research findings. Moreover, the application of weighted analysis corrects estimation biases arising from sampling errors, thereby further strengthening the accuracy and robustness of our results [13, 14].

All analyses accounted for the complex, multistage probability sampling design of NHANES by incorporating examination sample weights, strata, and primary sampling units (PSUs). Specifically, we used the mobile examination center (MEC) sample weights (WTMEC2YR) together with the design variables SDMVSTRA and SDMVPSU. For analyses combining two 2-year cycles (2011–2012 and 2013–2014), 4-year sample weights were constructed by dividing the 2-year MEC weights by 2, in accordance with NHANES analytic guidelines.

Multiple imputation

Multiple imputation is a well-established statistical approach that effectively addresses missing data issues by generating multiple plausible datasets, analyzing them separately, and combining the results to obtain valid estimates [15]. To assess the robustness of the complete-case analyses to missing covariate data, we performed multiple imputation by chained equations (MICE) under the assumption that the data were missing at random (MAR) [16]. The imputation model included the exposure variables, outcome variables, and all covariates used in the fully adjusted models. Five imputed datasets were generated, and each dataset was analyzed separately using the same modeling strategy as in the primary analysis. The resulting estimates were combined using Rubin's rules [17].

Propensity Score Matching

Propensity Score Matching (PSM) is a widely used statistical method in observational studies, primarily employed to reduce potential confounding bias [18]. Propensity score methods generally include four approaches: matching, stratification, weighting, and regression adjustment. To further reduce baseline imbalance between participants with and without sarcopenia, we performed 1:1 propensity score matching. Propensity scores were estimated using logistic regression based on the covariates included in the fully adjusted models [19]. Nearest-neighbor matching was performed on the logit of the propensity score, without replacement, using a caliper of 0.05. Sampling weights were incorporated into the propensity score estimation. After matching, survey-weighted analyses were repeated in the matched sample, and baseline characteristics were re-evaluated to assess covariate balance between the two groups [20].

Lagged Sensitivity Analysis

Lagged sensitivity analysis is a methodological approach used to reduce the risk of reverse causation by excluding early events that may be primarily driven by baseline illness or frailty rather than the exposure of interest. In this study, we applied a lagged sensitivity analysis by removing participants who died within the first two years of follow-up, under the rationale that such early deaths are more likely attributable to pre-existing health conditions. After excluding these participants, survey-weighted Cox proportional hazards models were re-estimated using the same covariate adjustment strategy as in the main analysis. This procedure ensured that the associations observed between exercise snacks and mortality were less likely to be confounded by early mortality related to underlying disease burden.

Handling of missing data and potential selection bias

The initial cohort comprised 19,931 participants, whereas the final complete-case analytical sample included 2,969 participants. Because this reduction may have introduced potential selection bias, we conducted additional sensitivity analyses to assess the robustness of the complete-case results. Given the structure of NHANES data collection, including the fact that some measurements (e.g., DXA scans) were obtained only in specific subsamples, the missing-data mechanism was considered likely to be compatible with a Missing at Random (MAR) assumption for the purpose of multiple imputation. Under this assumption, multiple imputation by chained equations (MICE) was performed as a sensitivity analysis. The results from the imputed data were broadly consistent with those from the complete-case analyses, supporting the robustness of the main findings. However, the possibility of residual selection bias due to missing or unavailable data cannot be entirely excluded.

REFERENCES

1. Nakayama JY, Mouhanna F, Whitfield GP, et al. Accelerometer-derived total activity and cardiometabolic health factors among U.S. adults, NHANES 2011–2014. Med Sci Sports Exerc. 2025; 57(12):2787–2794. doi:10.1249/MSS.0000000000003827
2. Aguiar EJ, Turner DT, Pleuss JD, Zheng P, Benitez CJ, Ducharme SW. Daily and peak monitor independent movement summary (MIMS) values associated with metabolic syndrome: NHANES 2011–12 and 2013–14. Scand J Med Sci Sports. 2024; 34(11):e14762. doi: 10.1111/sms.14762.
3. Rodríguez MÁ, Quintana-Cepedal M, Cheval B, Thøgersen-Ntoumani C, Crespo I, Olmedillas H. Effect of exercise snacks on fitness and cardiometabolic health in physically inactive individuals: systematic review and meta-analysis. Br J Sports Med. 2026; 60(2):133–141. doi: 10.1136/bjsports-2025-110027.
4. Islam H, Gibala MJ, Little JP. Exercise snacks: A novel strategy to improve cardiometabolic health. Exerc Sport Sci Rev. 2022; 50(1):31–37. doi: 10.1249/JES.0000000000000275.
5. Leroux A, Cui E, Smirnova E, Muschelli J, Schrack JA, Crainiceanu CM. NHANES 2011–2014: Objective physical activity is the strongest predictor of all-cause mortality. Med Sci Sports Exerc. 2024; 56(10):1926–1934. doi: 10.1249/MSS.0000000000003497.
6. Zheng P, Pleuss JD, Turner DS, Ducharme SW, Aguiar EJ. Dose–response association between physical activity (daily MIMS, peak 30-minute MIMS) and cognitive function among older adults: NHANES 2011–2014. J Gerontol A Biol Sci Med Sci. 2023; 78(2):286–291. doi: 10.1093/gerona/glac076.
7. Ducharme SW, Pleuss JD, Turner DS, Zheng P, Adandom II, Aguiar EJ. Normative peak physical activity values for monitor-independent movement summary units: National health and nutrition examination survey 2011–2014. J Phys Act Health. 2025; 22(10):1297–1306. doi: 10.1123/jpah.2025-0182.
8. Welk GJ, Lamoureux NR, Zeng C, et al. Equating NHANES monitor-based physical activity to self-reported methods to enhance ongoing surveillance efforts. Med Sci Sports Exerc. 2023; 55(6):1034–1043. doi: 10.1249/MSS.0000000000003123.
9. John D, Tang Q, Albinali F, Intille S. An open-source monitor-independent movement summary for accelerometer data processing. J Meas Phys Behav. 2019; 2(4):268–281. doi: 10.1123/jmpb.2018-0068.
10. Doncaster P, Spake R. Correction for bias in meta-analysis of little-replicated studies. Methods Ecol Evol. 2018; 9(3):634–644. doi: 10.1111/2041-210X.12927.
11. Cukier RI. Variance of a weighted mean force obtained using the weighted histogram analysis method. J Phys Chem B. 2013; 117(47):14785–14796. doi: 10.1021/jp407956c.
12. Beretta L, Tetek J. Better sum estimation via weighted sampling. ACM Trans Algorithms. 2021; 20:1–33. doi: 10.1145/3650030.
13. Markatou M. Mixture models, robustness, and the weighted likelihood methodology. Biometrics. 2000; 56(2):483–486. doi: 10.1111/j.0006-341X.2000.00483.x.
14. Cheah J, Roldán J, Ciavolino E, Ting H, Ramayah T. Sampling weight adjustments in partial least squares structural equation modeling: Guidelines and illustrations. Total Qual Manag Bus Excell. 2020; 32:1594–1613. doi: 10.1080/14783363.2020.1754 125.
15. Slade E, Naylor M. A fair comparison of tree-based and parametric methods in multiple imputation by chained equations. Stat Med. 2020; 39:1156–1166. doi: 10.1002/sim.8468.
16. Deng Y, Chang C, Ido M, Long Q. Multiple imputation for general missing data patterns in the presence of high-dimensional data. Sci Rep. 2016; 6:21689. doi: 10.1038/srep21689.
17. Resche-Rigon M, White I. Multiple imputation by chained equations for systematically and sporadically missing multilevel data. Stat Methods Med Res. 2018; 27:1634–1649. doi: 10.1177/0962280216666564.
18. Badhiwala J, Karmur B, Wilson J. Propensity score matching: A powerful tool for analyzing observational nonrandomized data. Clin Spine Surg. 2021; 34(1):22–24. doi: 10.1097/BSD.0000000000001055.
19. Schober P, Vetter T. Propensity score matching in observational research. Anesth Analg. 2020; 130(6):1616–1617. doi: 10.1213/ANE.0000000000004770.
20. Elze MC, Gregson J, Baber U, et al. Comparison of propensity score methods and covariate adjustment: evaluation in 4 cardiovascular studies. J Am Coll Cardiol. 2017 Jan 24; 69(3):345–357. doi: 10.1016/j.jacc.2016.10.060.

SUPPLEMENTARY TABLE S1. Characteristics of patients according to the all-cause mortality

Characteristic	All-cause mortality		
	**Survivor,** N = 2708	**Non- Survivor,** N = 261	***P* Value**
**Age (years)**	66.48 ± (7.71)	68.15 ± (7.55)	**0.038**
**Sex, n (%)**			0.6
Male	1,352 (51%)	143 (54%)	
Female	1,356 (49%)	118 (46%)	
**Race, n (%)**			**0.03**
Mexican American	350 (8.7%)	31 (7.9%)	
Other Hispanic	256 (5.8%)	15 (4.0%)	
Non-Hispanic White	1,093 (68%)	111 (68%)	
Non-Hispanic Black	558 (10%)	66 (13%)	
Other Race	451 (7.9%)	38 (7.1%)	
**Education, n (%)**			**0.048**
Less than 9th grade	156 (3.6%)	14 (4.6%)	
9–11th grade	342 (9.8%)	40 (14%)	
High school graduate	573 (21%)	70 (27%)	
Some college or AA degree	818 (30%)	77 (31%)	
College graduate or above	819 (35%)	60 (24%)	
**Marital, n (%)**			**0.02**
Married	1,621 (63%)	141 (58%)	
Widowed	53 (1.5%)	4 (2.7%)	
Divorced	384 (14%)	34 (13%)	
Separated	116 (3.0%)	13 (4.4%)	
Never married	348 (12%)	51 (18%)	
Living with partner	186 (6.1%)	18 (4.5%)	
**PIR, n (%)**			**0.002**
< 1.3	789 (19%)	104 (25%)	
1.3–3.5	867 (31%)	88 (41%)	
> 3.5	1,052 (49%)	69 (34%)	
**BMI (Kg/m^2)**	27.31 ± (5.82)	29.13 ± (6.43)	0.37
**Smoking, n (%)**			**0.010**
Never	1,560 (56%)	119 (47%)	
Former	534 (22%)	55 (18%)	
Current	614 (22%)	87 (35%)	
**Drinking, n (%)**			**0.002**
Never	630 (23.3%)	66 (25.3%)	
Former	232 (8.6%)	39 (14.9%)	
Light/Moderate	820 (30.3%)	73 (28.0%)	
Heavy	1026 (37.9%)	83 (31.8%)	
**Diabetes, n (%)**			**0.03**
Yes	281 (8%)	36 (11%)	
No	2,427 (92%)	225 (89%)	
**Hypertension, n (%)**			**0.010**
Yes	818 (29%)	102 (37%)	
No	1,890 (71%)	159 (63%)	
**Albumin (g/L)**	42.93 ± (3.03)	42.76 ± (3.66)	0.67
**Intake of protein (gm/d)**	70.51 ± (4.38)	70.19 ± (5.26)	0.54
**WBC (X10^9/L)**	7.23 ± (2.18)	6.99 ± (5.26)	**0.018**
**SII**	524 ± (276)	540 ± (317)	**< 0.001**
**Combined grip strength (kg)**	61.5 ± (18.3)	56.7 ± (17.9)	**0.09**
**Sarcopenia, n (%)**			**< 0.001**
Yes	362 (13%)	97 (35%)	
No	2,346 (87%)	164 (65%)	
**Total MIMS (unit/day)**	5,089.59 ± (1,924.93)	4,551.87 ± (2,272.50)	**0.024**
**MIMS-ES (unit/day)**	2,041.01 ± (756.28)	1,698.93 ± (943.86)	**0.005**
**Time-ES (min/day)**	86.53 ± (31.75)	73.09 ± (39.62)	**0.006**

mean ± standard deviation (SD) for continuous; n (%) for categorical; PIR, poverty income ratio; BMI, body mass index; WBC, White blood cell count; SII, systemic immune-inflammation index; MIMS, Monitor-Independent Movement Summary; MIMS- ES, daily average MIMS specific to exercise snacks; Time-ES, daily average time spent on exercise snacks.

SUPPLEMENTARY TABLE S2. Baseline characteristics of participants with and without sarcopenia after multiple imputation.

Characteristic	Overall<br>N = 19931	Non- Sarcopenia<br>N = 15272	Sarcopenia<br>N = 4659	P-Value
**Age (years)**	64.16 ± (8.2)	63.45 ± (7.4)	64.87 ± (9.1)	< **0.001**
**Sex, n (%)**				< **0.001**
Male	9,859 (49%)	6,882 (45%)	2,977 (64%)	
Female	10,072 (51%)	8,390 (55%)	1,682 (36%)	
**Race, n (%)**				< **0.001**
Mexican American	6,647 (33%)	5,357 (35%)	1,290 (28%)	
Other Hispanic	4,950 (25%)	3,952 (26%)	998 (21%)	
Non-Hispanic White	3,213 (16%)	2,584 (17%)	629 (14%)	
Non-Hispanic Black	3,085 (15%)	1,950 (13%)	1,135 (24%)	
Other Race	2,036 (10%)	1,429 (9.4%)	607 (13%)	
**Education, n (%)**				< **0.001**
Less than 9th grade	1,497 (7.5%)	1,103 (7.2%)	394 (8.5%)	
9–11th grade	2,648 (13%)	1,949 (13%)	699 (15%)	
High school graduate	4,283 (21%)	3,196 (21%)	1,087 (23%)	
Some college or AA degree	6,298 (32%)	4,857 (32%)	1,441 (31%)	
College graduate or above	5,205 (26%)	4,167 (27%)	1,038 (22%)	
**Marital, n (%)**				< **0.001**
Married	6,254 (31%)	5,638 (37%)	616 (13%)	
Widowed	908 (4.6%)	850 (5.6%)	58 (1.2%)	
Divorced	1,318 (6.6%)	1,190 (7.8%)	128 (2.7%)	
Separated	468 (2.3%)	407 (2.7%)	61 (1.3%)	
Never married	7,793 (39%)	5,208 (34%)	2,585 (55%)	
Living with partner	3,190 (16%)	1,979 (13%)	1,211 (26%)	
**PIR**	2.12 ± (1.61)	2.30 ± (1.65)	1.94 ± (1.56)	< **0.001**
**BMI (Kg/m²)**	25.2 ± (8.3)	26.1 ± (8.5)	22.9 ± (8.1)	< **0.001**
**Smoking, n (%)**				< **0.001**
Never	13,876 (70%)	10,115 (66%)	3,761 (81%)	
Former	2,996 (15%)	2,504 (16%)	492 (11%)	
Current	3,059 (15%)	2,653 (17%)	406 (8.7%)	
**Drinking, n (%)**				< **0.001**
Never	6,928 (34.7%)	5,153 (33.7%)	1,775 (38%)	
Former	11,170 (56%)	8,658 (56.6%)	2,512 (54%)	
Light/Moderate	825 (4.1%)	642(4.2%)	183(3.9%)	
Heavy	1008 (5.2%)	768 (5.5%)	240 (4.1%)	
**Diabetes, n (%)**				< **0.001**
Yes	1,822 (9%)	1,586 (10%)	186 (4%)	
No	18,159 (91%)	13,686 (90%)	4,473 (96%)	
**Hypertension, n (%)**				< **0.001**
Yes	4,287 (21%)	3,889 (25%)	398 (8.5%)	
No	15,644 (78%)	11,383 (75%)	4,261 (91%)	
**Albumin (g/L)**	43.65 ± (3.66)	44.69 ± (3.79)	43.33 ± (3.56)	< **0.001**
**Intake of protein (gm/d)**	72.34 ± (4.87)	73.87 ± (4.90)	71.87 ± (4.76)	< **0.001**
**WBC (X10⁹/L)**	7.17 ± (2.27)	7.03 ± (2.15)	7.63 ± (2.56)	< **0.001**
**Combined grip strength (kg)**	60.18 ± (18.4)	62.64 ± (18.8)	58.73 ± (18.2)	**0.024**
**MIMS (unit/day)**	4,956.73 ± (1,973.14)	4,987.40 ± (1,967.29)	4,856.21 ± (1,989.11)	**0.004**
**MIMS-ES (unit/day)**	2,026.79 ± (773.24)	2,050.98 ± (764.92)	2,019.42 ± (775.63)	**0.004**
**Time-ES (min/day)**	85.92 ± (34.14)	86.45 ± (35.42)	85.76 ± (33.74)	**0.011**
**SII**	476 ± (341)	430 ± (332)	490 ± (343)	< **0.001**

mean ± standard deviation (SD) for continuous; n (%) for categorical; PIR, poverty income ratio; BMI, body mass index; WBC, White blood cell count; SII, systemic immune-inflammation index; MIMS, Monitor-Independent Movement Summary; MIMS- ES, daily average MIMS specific to exercise snacks; Time-ES, daily average time spent on exercise snacks.

SUPPLEMENTARY TABLE S3. Association between exercise snacks and sarcopenia analyzed using logistic regression after multiple imputation.

	Model I		Model II		Model III	
	OR (95% CI)	*P* value	OR (95% CI)	*P* value	OR (95% CI)	*P* value
**MIMS (unit/day)**	0.96 (0.92, 0.99)	0.008	0.95 (0.90, 0.98)	0.006	0.90 (0.84, 0.96)	0.004
Q1	1.0		1.0		1.0	
Q2	0.74 (0.58, 0.89)	0.006	0.69 (0.56, 0.78)	0.005	0.67 (0.54, 0.84)	0.003
Q3	0.68 (0.53, 0.88)	0.005	0.62 (0.49, 0.79)	< 0.001	0.61 (0.47, 0.79)	< 0.001
Q4	0.63 (0.50, 0.80)	< 0.001	0.49 (0.40, 0.60)	< 0.001	0.44 (0.35, 0.56)	< 0.001
***P* for trend**		0.021		0.002		0.001
**MIMS-ES (unit/day)**	0.97 (0.93, 0.99)	0.008	0.96 (0.92, 0.98)	0.006	0.95 (0.90, 0.97)	0.005
Q1	1.0		1.0		1.0	
Q2	0.68 (0.53, 0.87)	0.005	0.63 (0.50, 0.79)	< 0.001	0.62 (0.49, 0.78)	< 0.001
Q3	0.61 (0.47, 0.78)	< 0.001	0.58 (0.44, 0.73)	< 0.001	0.49 (0.40, 0.60)	< 0.001
Q4	0.48 (0.39, 0.59)	< 0.001	0.47 (0.32,0.58)	< 0.001	0.43 (0.34, 0.55)	< 0.001
***P* for trend**		0.012		0.008		< 0.001
**Time-ES (min/day)**	0.95 (0.90, 0.99)	0.006	0.90 (0.86, 0.96)	0.004	0.89 (0.82,0.94)	0.002
Q1	1.0		1.0		1.0	
Q2	0.67 (0.52, 0.84)	0.003	0.64 (0.52, 0.81)	0.001	0.61 (0.47, 0.79)	< 0.001
Q3	0.62 (0.49, 0.79)	< 0.001	0.50 (0.45, 0.55)	< 0.001	0.46 (0.40, 0.54)	< 0.001
Q4	0.48 (0.44, 0.53)	0.004	0.44 (0.34, 0.56)	< 0.001	0.40 (0.32, 0.51)	< 0.001
***P* for trend**		0.002		0.001		< 0.001

CI, Confidence Interval; MIMS, Monitor-Independent Movement Summary; MIMS-ES, daily average MIMS specific to exercise snacks; Time-ES, daily average time spent on exercise snacks.
Model I was unadjusted for covariates;
Model II was adjusted for age, sex, race, marital status, educational level and poverty income ratio (PIR);
Model III was adjusted for age, sex, race, marital status, educational level and PIR, smoking status, alcohol consumption, hypertension, diabetes, albumin, intake of protein, WBC and SII.

SUPPLEMENTARY TABLE S4. Association of exercise snacks with all-cause mortality in participants from NHANES 2011–2014 after multiple imputation.

All-cause mortality	Model I		Model II		Model III	
	HR (95% CI)	*P* value	HR (95% CI)	*P* value	HR (95% CI)	*P* value
**MIMS (unit/day)**	0.91 (0.80, 0.98)	0.009	0.90 (0.78, 0.97)	0.007	0.89 (0.76, 0.96)	0.002
Q1	1.0		1.0		1.0	
Q2	0.81 (0.76, 0.86)		0.79 (0.75, 0.85)		0.73 (0.66, 0.82)	
Q3	0.70 (0.59, 0.84)		0.65 (0.56, 0.74)		0.63 (0.55, 0.71)	
Q4	0.61 (0.48, 0.72)		0.57 (0.48, 0.69)		0.54 (0.41, 0.67)	
***P* for trend**		0.025		0.013		0.007
**MIMS-ES (unit/day)**	0.89 (0.78, 0.96)	0.002	0.84 (0.76, 0.90)	0.001	0.83 (0.74, 0.89)	0.001
Q1	1.0		1.0		1.0	
Q2	0.78 (0.67, 0.87)		0.72 (0.61, 0.85)		0.71 (0.59, 0.82)	
Q3	0.70 (0.61, 0.80)		0.66 (0.57, 0.76)		0.65 (0.53, 0.73)	
Q4	0.63 (0.56, 0.72)		0.58 (0.47, 0.71)		0.54 (0.42, 0.63)	
***P* for trend**		0.026		0.016		0.008
**Time-ES (min/day)**	0.88 (0.82, 0.95)	< 0.001	0.84 (0.78, 0.92)	< 0.001	0.82 (0.74, 0.90)	< 0.001
Q1	1.0		1.0		1.0	
Q2	0.79 (0.55, 0.92)	< 0.001	0.73 (0.62, 0.87)	< 0.001	0.70 (0.59, 0.85)	< 0.001
Q3	0.65 (0.57, 0.75)	< 0.001	0.64 (0.54, 0.78)	< 0.001	0.63 (0.56, 0.72)	< 0.001
Q4	0.48 (0.31, 0.65)	< 0.001	0.45 (0.28, 0.62)	< 0.001	0.43 (0.25, 0.52)	< 0.001
***P* for trend**		0.007		0.006		0.001

MIMS, Monitor-Independent Movement Summary; MIMS- ES, daily average MIMS specific to exercise snacks; Time-ES, daily average time spent on exercise snacks.
Model I was unadjusted for covariates;
Model II was adjusted for age, sex, race, marital status, educational level and poverty income ratio (PIR);
Model III was adjusted for age, sex, race, marital status, educational level and PIR, smoking status, alcohol consumption, hypertension, diabetes, combined grip strength, albumin, intake of protein, WBC and SII.

SUPPLEMENTARY TABLE S5. Baseline characteristics of participants with and without sarcopenia after PSM

Characteristic	Overall N = 858	Non- Sarcopenia N = 429	Sarcopenia N = 429	*P*-value	SMD
**Age (years)**	65.75 ± (8.66)	65.88 ± (7.17)	65.62 ± (9.92)	0.390	0.093
**Sex, n (%)**				0.592	0.019
Male	432 (53%)	214 (51%)	218 (54%)		
Female	426 (47%)	215 (49%)	211 (46%)		
**Race, n (%)**				0.437	0.076
Mexican American	172 (15%)	87 (15%)	85 (15%)		
Other Hispanic	111 (9.1%)	65 (11%)	46 (7.6%)		
Non-Hispanic White	299 (59%)	144 (58%)	155 (61%)		
Non-Hispanic Black	138 (8.8%)	69 (9.2%)	69 (8.4%)		
Other Race	138 (8.1%)	64 (7.8%)	74 (8.4%)		
**Education, n (%)**				0.805	0.035
Less than 9th grade	83 (6.6%)	47 (7.2%)	36 (6.0%)		
9–11th grade	118 (11%)	56 (11%)	62 (11%)		
High school graduate	187 (22%)	93 (24%)	94 (21%)		
Some college or AA degree	259 (32%)	127 (31%)	132 (33%)		
College graduate or above	211 (28%)	106 (27%)	105 (30%)		
**Marital, n (%)**				0.769	0.097
Married	488 (60%)	236 (62%)	252 (58%)		
Widowed	14 (1.5%)	8 (1.0%)	6 (1.9%)		
Divorced	99 (11%)	46 (10%)	53 (13%)		
Separated	45 (3.8%)	25 (3.4%)	20 (4.1%)		
Never married	139 (15%)	72 (15%)	67 (16%)		
Living with partner	73 (8.2%)	42 (9.0%)	31 (7.3%)		
**PIR, n (%)**				0.859	0.037
< 1.3	315 (26%)	160 (26%)	155 (26%)		
1.3–3.5	290 (35%)	146 (34%)	144 (36%)		
> 3.5	253 (39%)	123 (40%)	130 (38%)		
**BMI (Kg/m^2)**	31.23 ± (6.26)	31.31 ± (6.40)	31.15 ± (6.13)	0.872	0.007
**Smoking, n (%)**				0.647	0.035
Never	528 (61%)	262 (60%)	266 (61%)		
Former	152 (18%)	75 (20%)	77 (17%)		
Current	178 (21%)	92 (20%)	86 (22%)		
**Drinking, n (%)**				0.602	0.076
Never	274 (32%)	143 (33.3%)	131 (30.5%)		
Former	90 (10%)	47 (11%)	43 (10%)		
Light/Moderate	196 (22.8%)	81 (18.8%)	115 (26.8%)		
Heavy	298 (35.2%)	158 (36.9%)	140 (32.7%)		
**Diabetes, n (%)**				0.704	0.007
Yes	103 (10%)	52 (9.6%)	51 (10%)		
No	755 (90%)	377 (90%)	378 (90%)		
**Hypertension, n (%)**				0.726	< 0.001
Yes	282 (30%)	141 (29%)	141 (31%)		
No	576 (70%)	288 (71%)	288 (69%)		
**Albumin (g/L)**	42.71 ± (3.10)	42.94 ± (3.00)	42.48 ± (3.19)	0.161	0.024
**Intake of protein (gm/d)**	70.82 ± (4.40)	71.06 ± (4.44)	70.59 ± (4.35)	0.079	0.052
**WBC (X10^9/L)**	7.33 ± (2.18)	7.18 ± (2.14)	7.48 ± (2.22)	0.275	0.081
**SII**	531.99 ± (276.36)	515.01 ± (277.05)	548.75 ± (274.97)	0.092	0.084
**Combined grip strength (kg)**	62.98 ± (18.84)	63.81 ± (18.52)	62.17 ± (19.14)	0.208	0.042
**MIMS (unit/day)**	4,761.29 ± (1,891.61)	4,784.38 ± (1,948.95)	4,737.88 ± (1,835.23)	0.906	0.029
**MIMS-ES (unit/day)**	1,890.59 ± (796.81)	1,896.64 ± (765.99)	1,884.62 ± (826.95)	0.965	0.087
**Time-ES (min/day)**	69.87 ± (33.83)	72.28 ± (30.83)	67.50 ± (36.43)	0.023	0.029

mean ± standard deviation (SD) for continuous; n (%) for categorical; PIR, poverty income ratio; BMI, body mass index; WBC, White blood cell count; SII, systemic immune-inflammation index; MIMS, Monitor-Independent Movement Summary; MIMS- ES, daily average MIMS specific to exercise snacks; Time-ES, daily average time spent on exercise snacks.

SUPPLEMENTARY TABLE S6. Association between exercise snacks and sarcopenia analyzed using logistic regression after PSM

	Model I		Model II		Model III	
	**OR (95% CI)**	***P* value**	**OR (95% CI)**	***P* value**	**OR (95% CI)**	***P* value**
**MIMS (unit/day)**	0.97 (0.94, 0.99)	0.015	0.96 (0.94, 0.98)	0.009	0.95 (0.91, 0.98)	0.006
Q1	1.0		1.0		1.0	
Q2	0.96 (0.92, 0.98)	0.018	0.93 (0.88, 0.96)	0.008	0.91 (0.85, 0.95)	0.006
Q3	0.93 (0.85, 0.97)	0.009	0.91 (0.85, 0.95)	0.007	0.88 (0.81, 0.93)	0.005
Q4	0.89 (0.76, 0.96)	0.008	0.85 (0.75, 0.92)	0.006	0.84 (0.79, 0.92)	0.005
***P* for trend**		0.027		0.025		0.029
**MIMS-ES (unit/day)**	0.96 (0.92, 0.99)	0.016	0.94 (0.89, 0.98)	0.015	0.92 (0.85, 0.96)	0.009
Q1	1.0		1.0		1.0	
Q2	0.95 (0.91, 0.99)	0.018	0.93 (0.85, 0.98)	0.007	0.92 (0.87, 0.97)	0.007
Q3	0.89 (0.81, 0.98)	0.005	0.87 (0.76, 0.94)	0.006	0.86 (0.81, 0.96)	0.005
Q4	0.88 (0.73, 0.96)	0.004	0.84 (0.76,0.92)	0.001	0.82 (0.69, 0.89)	< 0.001
***P* for trend**		0.018		0.021	0.025	
**Time-ES (min/day)**	0.96 (0.92, 0.99)	0.019	0.93 (0.88, 0.98)	0.016	0.91 (0.88, 0.96)	0.008
Q1	1.0		1.0		1.0	
Q2	0.95 (0.89, 0.98)	0.028	0.92 (0.87, 0.97)	0.018	0.90 (0.84, 0.96)	0.017
Q3	0.89 (0.76, 0.97)	0.015	0.88 (0.79, 0.96)	0.008	0.86 (0.74, 0.95)	0.006
Q4	0.88 (0.77, 0.96)	0.008	0.84 (0.79, 0.91)	0.006	0.83 (0.74, 0.93)	0.005
***P* for trend**		0.003		0.007	0.015	

PSM, Propensity Score Matching; CI, Confidence Interval; MIMS, Monitor-Independent Movement Summary; MIMS-ES, daily average MIMS specific to exercise snacks; Time-ES, daily average time spent on exercise snacks.
Model I was unadjusted for covariates;
Model II was adjusted for age, sex, race, marital status, educational level and poverty income ratio (PIR);
Model III was adjusted for age, sex, race, marital status, educational level and PIR, smoking status, alcohol consumption, hypertension, diabetes, albumin, intake of protein, WBC and SII.

SUPPLEMENTARY TABLE S7. Association of exercise snacks with all-cause mortality in participants from NHANES 2011–2014 after PSM

All-cause mortality	Model I		Model II		Model III	
	HR (95% CI)	*P* value	HR (95% CI)	*P* value	HR (95% CI)	*P* value
**MIMS (unit/day)**	0.89 (0.81, 0.98)	0.009	0.87 (0.78, 0.96)	0.007	0.84 (0.76, 0.95)	0.003
Q1	1.0		1.0		1.0	
Q2	0.73 (0.60, 0.86)		0.71 (0.59, 0.84)		0.70 (0.39, 0.82)	
Q3	0.70 (0.61, 0.82)		0.70 (0.58, 0.81)		0.67 (0.55, 0.79)	
Q4	0.48 (0.31, 0.73)		0.47 (0.31, 0.71)		0.47 (0.32, 0.71)	
***P* for trend**		0.0417		0.0269		0.0175
**MIMS-ES (unit/day)**	0.87 (0.79, 0.98)	0.007	0.85 (0.78, 0.95)	0.006	0.81 (0.71, 0.91)	0.002
Q1	1.0		1.0		1.0	
Q2	0.55 (0.44, 0.78)		0.52 (0.43, 0.72)		0.51 (0.43, 0.71)	
Q3	0.40 (0.23, 0.60)		0.39 (0.23, 0.57)		0.39 (0.22, 0.59)	
Q4	0.39 (0.25, 0.51)		0.37 (0.24, 0.47)		0.37 (0.23, 0.48)	
***P* for trend**		0.0336		0.0206		0.0158
**Time-ES (min/day)**	0.87 (0.74, 0.92)	< 0.001	0.83 (0.72, 0.91)	< 0.001	0.80 (0.67, 0.89)	< 0.001
Q1	1.0		1.0		1.0	
Q2	0.50 (0.43, 0.74)	< 0.001	0.50 (0.42, 0.67)	< 0.001	0.50 (0.39, 0.66)	< 0.001
Q3	0.49 (0.33, 0.64)	0.002	0.49 (0.32, 0.64)	0.004	0.48 (0.31, 0.63)	0.005
Q4	0.42 (0.24, 0.63)	< 0.001	0.43 (0.26, 0.58)	< 0.001	0.42 (0.25, 0.56)	< 0.001
***P* for trend**		0.0170	0.0160		0.0137	

MIMS, Monitor-Independent Movement Summary; MIMS- ES, daily average MIMS specific to exercise snacks; Time-ES, daily average time spent on exercise snacks.
Model I was unadjusted for covariates;
Model II was adjusted for age, sex, race, marital status, educational level and poverty income ratio (PIR);
Model III was adjusted for age, sex, race, marital status, educational level and PIR, smoking status, alcohol consumption, hypertension, diabetes, combined grip strength, albumin, intake of protein, WBC and SII.

SUPPLEMENTARY TABLE S8. Hazard ratios for all-cause mortality according to exercise snacks in lagged sensitivity analyses.

All-cause mortality	Model I		Model II		Model III	
	HR (95% CI)	*P* value	HR (95% CI)	*P* value	HR (95% CI)	*P* value
**MIMS (unit/day)**	0.95 (0.89, 0.98)	< 0.001	0.93 (0.87, 0.97)	0.016	0.91 (0.85, 0.96)	0.018
Q1	1.0		1.0		1.0	
Q2	0.86 (0.72, 0.97)		0.87 (0.76, 0.98)		0.88 (0.73, 0.98)	
Q3	0.84 (0.74, 0.91)		0.81 (0.73, 0.92)		0.77 (0.69, 0.85)	
Q4	0.77 (0.65, 0.89)		0.76 (0.67, 0.84)		0.72 (0.64, 0.81)	
***P* for trend**		0.015		0.012		0.008
**MIMS-ES (unit/day)**	0.95 (0.89, 0.98)	0.007	0.92 (0.85, 0.97)	0.006	0.91 (0.82, 0.96)	0.002
Q1	1.0		1.0		1.0	
Q2	0.89 (0.75, 0.96)		0.87 (0.74, 0.94)		0.83 (0.76, 0.91)	
Q3	0.83 (0.72, 0.94)		0.81 (0.73, 0.91)		0.78 (0.64, 0.89)	
Q4	0.75 (0.34, 0.88)		0.73 (0.65, 0.82)		0.72 (0.64, 0.85)	
***P* for trend**		0.024		0.013	0.006	
**Time-ES (min/day)**	0.94 (0.89, 0.97)	0.012	0.93 (0.88, 0.96)	< 0.001	0.90 (0.85, 0.95)	< 0.001
Q1	1.0		1.0		1.0	
Q2	0.89 (0.73, 0.96)	0.002	0.87 (0.80, 0.94)	0.004	0.84 (0.76, 0.94)	0.005
Q3	0.86 (0.74, 0.93)	< 0.001	0.83 (0.76, 0.92)	< 0.001	0.82 (0.75, 0.93)	< 0.001
Q4	0.76 (0.69, 0.84)	< 0.001	0.73 (0.67, 0.81)	< 0.001	0.68 (0.59, 0.76)	< 0.001
***P* for trend**		0.013		0.007		0.004

MIMS, Monitor-Independent Movement Summary; MIMS-ES, daily average MIMS specific to exercise snacks; Time-ES, daily average time spent on exercise snacks.
Model I was unadjusted for covariates;
Model II was adjusted for age, sex, race, marital status, educational level and poverty income ratio (PIR);
Model III was adjusted for age, sex, race, marital status, educational level and PIR, smoking status, alcohol consumption, hypertension, diabetes, albumin, intake of protein, WBC and SII.

SUPPLEMENTARY TABLE S9. Additional model diagnostics for the fully adjusted main regression models.

Outcome	Exposure variable	Model	Diagnostic 1	Diagnostic 2
**Sarcopenia** (Weighted logistic regression)	MIMS	Model III	R^2=0.046	AIC: 28.88
	MIMS-ES		R^2=0.054	AIC: 28.84
	Time-ES		R^2=0.117	AIC: 27.98
**Combined grip strength** (Weighted linear regression)	MIMS	Model III	Adjusted R^2=0.038	RMSE:18.89
	MIMS-ES		Adjusted R^2=0.040	RMSE:18.19
	Time-ES		Adjusted R^2=0.043	RMSE:18.08

R^2 and AIC are reported for the fully adjusted logistic regression models of sarcopenia. Adjusted R^2 and RMSE are reported for the fully adjusted linear regression models of combined grip strength. Higher R^2 or adjusted R^2 values indicate greater explained variance, whereas lower AIC or RMSE values indicate better relative model performance among comparable models.

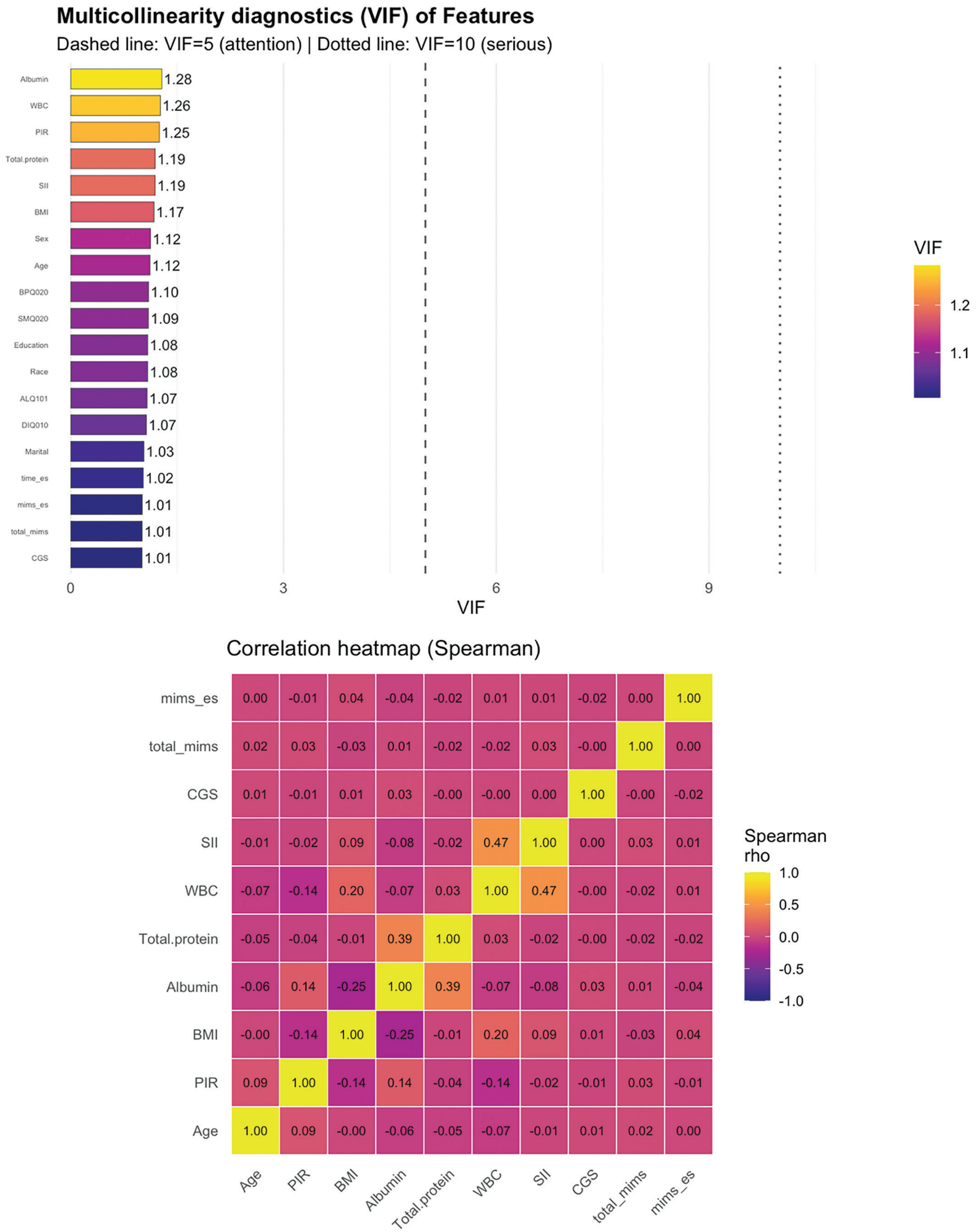

SUPPLEMENTARY FIG. S1. Multicollinearity diagnostics (VIF) of features and Spearman's correlation test among selected features.

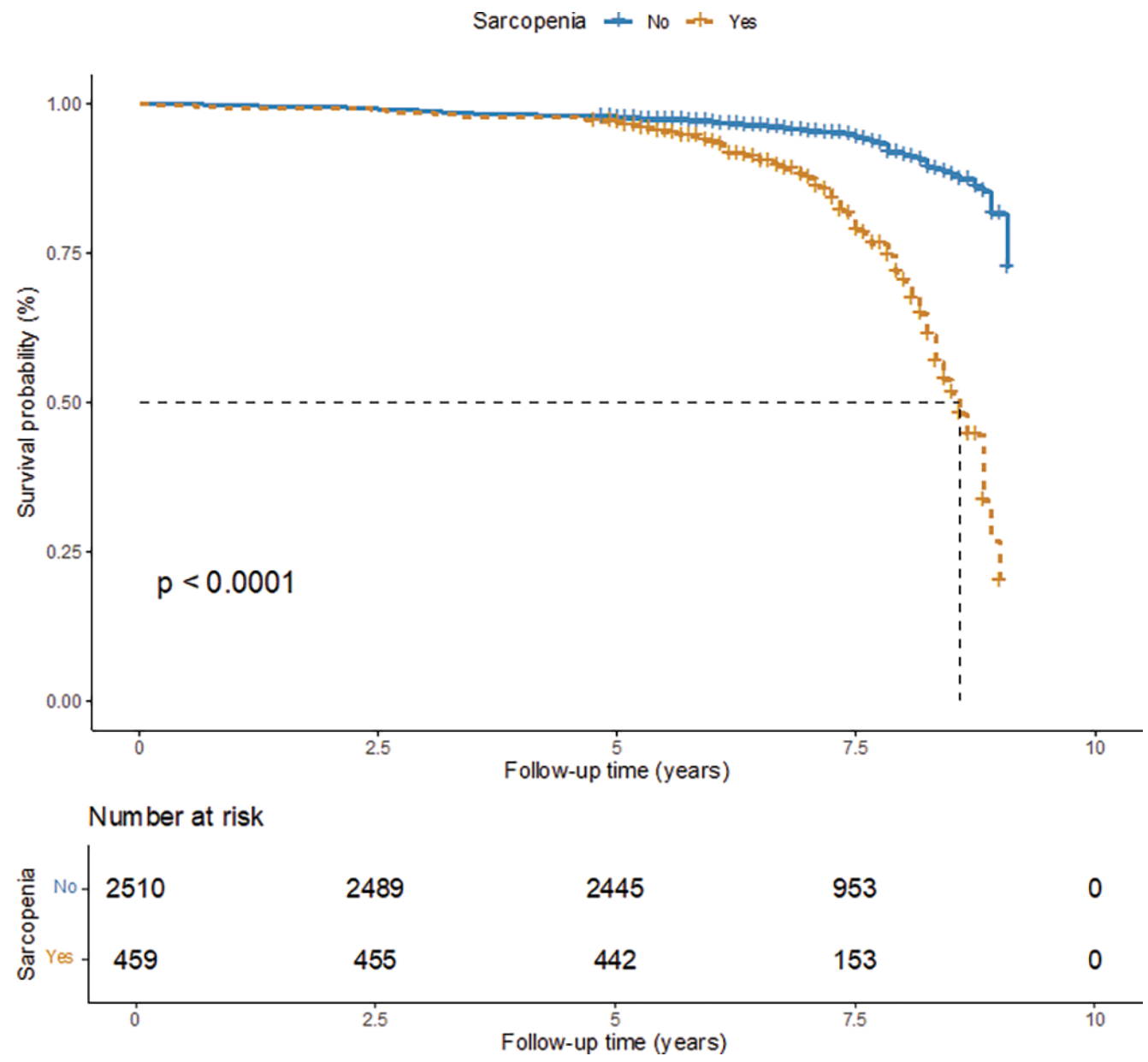

SUPPLEMENTARY FIG. S2. Kaplan–Meier survival curves for all-cause mortality according to sarcopenia status.

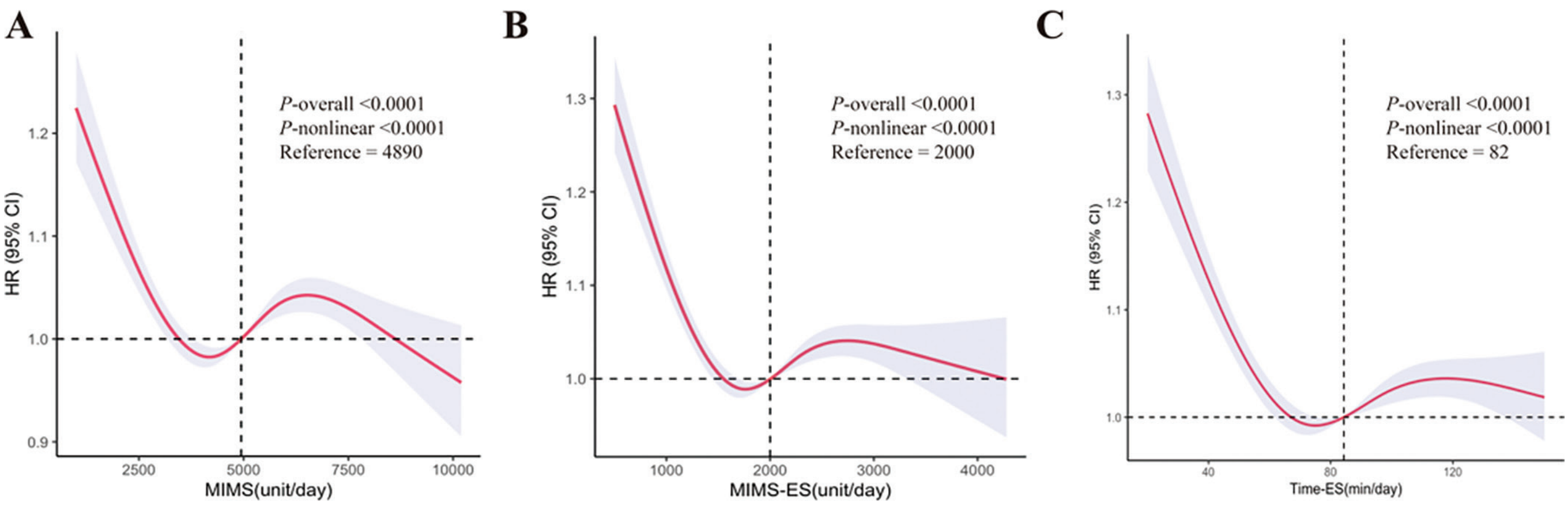

SUPPLEMENTARY FIG. S3. RCS curve of the relationship between exercise snacks and all-cause mortality.
MIMS, Monitor-Independent Movement Summary; MIMS- ES, daily average MIMS specific to exercise snacks; Time-ES, daily average time spent on exercise snacks; RCS, restricted cubic spline.
